# Non-canonical translation initiation of the spliced mRNA encoding the human T-cell leukemia virus type 1 basic leucine zipper protein

**DOI:** 10.1093/nar/gky802

**Published:** 2018-09-12

**Authors:** C Joaquín Cáceres, Jenniffer Angulo, Fernando Lowy, Nataly Contreras, Beth Walters, Eduardo Olivares, Delphine Allouche, Anne Merviel, Karla Pino, Bruno Sargueil, Sunnie R Thompson, Marcelo López-Lastra

**Affiliations:** 1 Departamento de Enfermedades Infecciosas e Inmunología Pediátrica, Laboratorio de Virología Molecular, Instituto Milenio de Inmunología e Inmunoterapia, Centro de Investigaciones Médicas, Escuela de Medicina, Pontificia Universidad Católica de Chile, Marcoleta 391, Santiago, Chile; 2 Department of Microbiology, University of Alabama at Birmingham, Birmingham AL 35294, USA; 3 CNRS UMR 8015, Laboratoire de cristallographie et RMN Biologique, Université Paris Descartes, 4 avenue de l’Observatoire, 75270 Paris Cedex 06, France

## Abstract

Human T-cell leukemia virus type 1 (HTLV-1) is the etiological agent of adult T-cell leukemia (ATL). The HTLV-1 basic leucine zipper protein (HBZ) is expressed in all cases of ATL and is directly associated with virus pathogenicity. The two isoforms of the HBZ protein are synthesized from antisense messenger RNAs (mRNAs) that are either spliced (*sHBZ*) or unspliced (*usHBZ*) versions of the *HBZ* transcript. The *sHBZ* and *usHBZ* mRNAs have entirely different 5′untranslated regions (5′UTR) and are differentially expressed in cells, with the sHBZ protein being more abundant. Here, we show that differential expression of the HBZ isoforms is regulated at the translational level. Translation initiation of the *usHBZ* mRNA relies on a cap-dependent mechanism, while the *sHBZ* mRNA uses internal initiation. Based on the structural data for the sHBZ 5′UTR generated by SHAPE in combination with 5′ and 3′ deletion mutants, the minimal region harboring IRES activity was mapped to the 5′end of the sHBZ mRNA. In addition, the sHBZ IRES recruited the 40S ribosomal subunit upstream of the initiation codon, and IRES activity was found to be dependent on the ribosomal protein eS25 and eIF5A.

## INTRODUCTION

The human T-cell leukemia virus type 1 (HTLV-1), a member of the *Retroviridae* family, was the first retrovirus to be associated with a disease in humans. After a prolonged latency period, ∼4% of HTLV-1 infected individuals develop adult T-cell leukemia (ATL), an aggressive CD4 T-cell derived leukemia, with an extremely poor prognosis (1). Among the viral proteins that are expressed during infection, the HTLV-1 basic leucine zipper protein (HBZ) is consistently found in infected cells of ATL patients but not in those of asymptomatic HTLV-1 carriers, suggesting that the protein plays a key role in HTLV-1 pathogenesis (2,3). HTLV-1 synthesizes two different isoforms of the HBZ protein, which are encoded by two different capped and polyadenylated antisense transcripts. The transcripts have different transcriptional start sites and one undergoes splicing, resulting in either the spliced (*sHBZ*) or unspliced (*usHBZ*) messenger RNAs (mRNAs) (4). The *sHBZ* and *usHBZ* mRNAs that encode for the HBZ isoforms, sHBZ and usHBZ, have entirely different 5′untranslated regions (5′UTRs). The HBZ isoforms are 95% identical and differ only by seven amino acids at their N-terminus. Both HBZ isoforms exert similar but not identical roles in HTLV-1 infectivity, persistence and oncogenesis (2,3). HBZ transcripts have been detected in ATL cell lines and in primary cells harvested from patients with acute ATL; however, the sHBZ protein is more abundant in cells than the usHBZ (4–8). A study shows that the expression of the *sHBZ* mRNA is higher (4-fold) than that of the *usHBZ* mRNA in HTLV-1 infected cells recovered from ATL patients (7). These results contrast with another dataset showing that the level of both the *sHBZ* and *usHBZ* transcripts are similar in ATL cell lines and in primary cells harvested from ATL-patients (4). *In vitro* experiments in which the HBZ proteins were expressed from a plasmid in the absence of other viral proteins have suggested that differences in protein expression levels are translationally regulated (5). These results suggest that differences in translation of the isoforms of the HBZ mRNAs are due to the differences in the 5′UTRs.

In eukaryotes, the 5′UTR is important for regulating translation initiation, which is the rate-limiting step in protein synthesis (9). Translation initiation for most eukaryotic mRNAs occurs through a cap-dependent mechanism in which the 5′cap structure is recognized by the eukaryotic initiation factor (eIF) 4F complex that is composed of the cap-binding protein (eIF4E), the scaffolding protein (eIF4G) and the RNA helicase (eIF4A) (9). The 40S ribosomal subunit is recruited to the mRNA as part of the 43S preinitiation complex (PIC), consisting of the 40S ribosomal subunit, eIF1, eIF1A, eIF3, eIF5 and the ternary complex (eIF2, initiator Met-transfer RNA (tRNA)_i_^Met^ and GTP). The PIC is recruited to the mRNA through an interaction between eIF3 and eIF4F. Upon recruitment to the mRNA, the 40S scans in a 5′ to 3′ direction until the start codon is recognized followed by 60S subunit joining to form the 80S ribosome (9). In some cases, specialized elements or RNA structures such as internal ribosome entry sites (IRESs) can directly recruit the 40S ribosomal subunit internally to the mRNA in a cap-independent fashion allowing for a non-canonical mode of translation initiation (10). IRES-mediated translation initiation often occurs under physiological conditions when cap-dependent translation initiation is inhibited (11,12).

Intrigued by the possibility that differential HBZ isoform expression results from differences in the translational efficiencies or modes of translation of the *sHBZ* and *usHBZ* mRNAs, we examined translation of the *sHBZ* and *usHBZ* mRNAs in a virus free context using monocistronic and bicistronic reporters both *in vitro* and in cells. Our results show that the *sHBZ* mRNA uses a non-canonical scanning-dependent mechanism whereby the 40S subunit is recruited internally to the 5′UTR upstream of the start codon. In contrast, the *usHBZ* mRNA appears to require a 5′cap to recruit the 40S ribosomal subunit. Since the sHBZ 5′UTR resulted in higher levels of protein expression relative to the usHBZ 5′UTR, this suggests that translation of the *usHBZ* mRNA is repressed since cap-dependent translation is usually more efficient than cap-independent initiation. These findings demonstrate that the HTLV-1 HBZ isoform levels are regulated at the step of translation initiation.

## MATERIALS AND METHODS

### Plasmids and template DNA

The dl ΔEMCV (13,14), dl HTLV-1 IRES (15), dl HCV 1b (14) (herein referred to as dl HCV IRES), dl PV IRES (13) and dl HIV-1 IRES (13) were previously described. The RLuc (16) and FLuc (17) (herein referred to as Glo-FLuc) vectors were kindly provided by Dr Ricardo Soto-Rifo (Universidad de Chile, Chile). Plasmids expressing the functional wild-type (genotype A16) or an inactive mutant human rhinoviruses (HRV) 2A protease (18,19), herein referred to as p2A-wt and p2A-mut were generously supplied by Dr Ann C. Palmenberg and Dr Kelly Watters (University of Wisconsin-Madison, USA). The DNA sequence that corresponds to the 5′UTR of the spliced (s) version and the unspliced (us) version of *HBZ* mRNA were recovered from the HTLV-1 K30 provirus (accession number: L03561), kindly provided by Dr B. Barbeau, (Universite du Québec, Canada) (20). Unless specified, enzymes (catalog number is indicated) were purchased from ThermoFisher Scientific (Waltham, MA, USA). To generate the monocistronic (m) plasmid msFLuc, the sHBZ 5′UTR was recovered using primers F-HindIII-T7-Sp (5′-AAGCTTTAATACGACTCACTATAGGCCGGCAGTCAGTCGTGAAACAAAGGG-3′) and EcoRI-SpR (5′-TTCCATGAATTCCCACGCCGGTTGAG-3′). To construct the musFLuc plasmid, the usHBZ 5′UTR was obtained with primers F-HindIII-T7-Us (5′-AAGCTTTAATACGACTCACTATAGGGTAACGCGTCCATCG-3′) and R-EcoRI-Us (5′-CCATGAATTCGCTTATTATCAGCCCACTTCCC-3′). Amplicons were digested with HindIII (#ER0591) and EcoRI (#ER0271), and cloned upstream from the firefly luciferase (FLuc) open reading frame (ORF) of the dl ΔEMCV vector previously digested with the same enzymes. Digestion of dl ΔEMCV with HindIII and EcoRI eliminates the *Renilla* luciferase (RLuc) ORF. The musFLucAAC monoscitronic plasmid was generated by overlapping polymerase chain reaction (PCR) using the *Pfu* DNA Polymerase (#EP0571) according to the manufacturer’s instructions (21). Briefly, an amplicon generated from musFluc using primers F-HindIII-Us4ATG-(5′-CTTTTGCAAAAAGCTTTAATACGACTCACTATAGG-3′) and R-OvUs4ATG-(5′-GGACGCGTTATCGGCTCAGCTCTACAGTTC-3′), together with the amplicon generated from an us-4ATG-Oligo synthetic oligonucleotide (5′-TAACGCGTCCATCG**AAC**GGGTCCCAGGTGATCTG**AAC**CTCTGGACAGGTGGCCAGTAGGGCGTGACG**AAC**TAGGCGGGCCGAACATAGTCCCCCAGAG**AAC**GGGCACCAGTCGCCTTGTACACAGTCTCCAAACACGTAGACTGGGTATCCGAAAAGAAGACTCTGTCTAAACCCTGGGAAGTGGGCTGATAAT-3′) amplified using primers F-Oligous4ATG-(5′-GCTGAGCCGATAACGCGTCCATCGAACG-3′) and R-EcoRI-us, was used in an overlapping PCR. The final PCR product was digested with HindIII and EcoRI and cloned into the musFLuc plasmid backbone lacking the usHBZ 5′UTR sequence. To generate the msFLucAAC plasmid, the sHBZ 5′UTR was amplified using primers HindIII-T7Sp-F-AAC (5′CAAAAAGCTTTAATACGACTCACTATAGGCCGGCAGTCAGTCGTGAAACAAAGGG-3′) and EcoRI-SpR (5′-TTCCATGAATTCCCACGCCGGTTGAG-3′). The amplicon was digested with HindIII and EcoRI and cloned into the msFLuc vector backbone lacking the sHBZ 5′UTR sequence. In plasmids musFLucCUC, musFLucAAC/CUC, msFLucCUC and msFLucAAC/CUC, the FLuc initiation codon (AUG) was mutated to CUC by PCR using the Phusion High Fidelity DNA Polymerase (#F530S) following the manufacturer’s instructions and primers CUC_Fluc_musHBZ_For (5′-ATAATAAGCGAATTC**C**T**C**GAAGACGCC-3′) and RV_4R (5′-GACGATAGTCATGCCCCGCG-3′) for musFLuc mutants, and primers CUC_Fluc_mspHBZ_For (5′-GGCGTGGGAATTC**C**T**C**GAAGACGC-3′) and RV_4R for the msFLuc mutants. The amplicons (2008 and 2006 bp, from musFluc and msFluc, respectively) were resolved in by agarose (1%) gel electrophoresis and purified using the Kit Wizard^®^ SV Gel and PCR Clean-Up System (#A9282; Promega, Madison, WI, USA). The purified amplicons were quantified by spectrophotometry (Nanophotometer, Implen Inc., Westlake Village, CA, USA). Both amplicons were digested with EcoRI and XbaI (#ER0685) restriction enzymes and cloned into musFLuc, musFlucAAC, msFLuc and msFlucAAC DNAs previously digested with EcoRI and XbaI. The entire FLuc ORF was replaced by the amplicons. To generate the dual luciferase (dl) bicistronic vectors, dl sHBZ 5′UTR and dl usHBZ 5′UTR, the sHBZ 5′UTR or usHBZ 5′UTR was recovered by PCR using primers FEcoRI-dualSp (5′-GAATCCCCGGCAGTCAGTCGTGAATG-3′) and RNcoI-dualSp (5′-CCATGGCCACGCCGGTGGAGTCGC-3′) from the sHBZ 5′UTR DNA or primer FEcoRI-dualUs (5′-GAATTCGGGGTAGGACCTTGAGG-3′) and RNcoI-dualUs (5′-CCATGGGCTTATTATCAGCCCACTTC-3′) from the usHBZ 5′UTR DNA. The amplicons were digested using EcoRI and NcoI (#ER0575) and cloned in the intercistronic space of the dl HTLV-1 IRES previously digested with EcoRI*/*NcoI and NcoI*/*XbaI as in (15). The dl sHBZ 5′UTR_CUC (initiation codon of FLuc mutated to CUC) was generated by overlapping PCR using the Phusion High Fidelity DNA Polymerase. The 5′ fragment (837 bp) was amplified using the primers RlucNic1: (5′-TCAAATCGTTCGTTGAGCGAGTTC-3′) and O_CUC_Fluc_dlHBZ_Rev (5′-GGCGTCTTC**G**A**G**GGCCACG-3′). The 3′ fragment (2000 bp) was amplified using the primers O_CUC_Fluc_dlHBZ_For (5′-CGTGGCC**C**T**C**GAAGACGCC-3′) and RV_4R. The amplicons were resolved on agarose (1%) gels, purified using the Kit Wizard^®^ SV Gel and PCR Clean-Up System and quantified by spectrophotometry (Implen Nanophotometer). The same number of copies of both amplicons were used in an overlapping PCR with primers RlucNic1 and RV_4R. The final amplicon was digested with XhoI (#ER0691) and XbaI restriction enzymes and cloned into the dl sHBZ 5′UTR plasmid backbone digested with the same restriction enzymes. For the promoterless vector ΔSV40 dl sHBZ 5′UTR, the dl sHBZ 5′UTR plasmid was digested with MluI (#ER0561) and Eco147I (*StuI*) (#ER0421), and religated. For the generation of dl ΔΔEMCV sHBZ 5′UTR, the dl sHBZ 5′UTR was digested with EcoRI and XhoI and religated. The dl 40–261, dl 64–264 and dl 79–261 were obtained by PCR using the *Pfu* DNA polymerase with the using primers SpJC1EcoRI-F (5′-AGGGGGAATTCTTTCGATCTGTAACGGCGCAG-3′), SpJC2EcoRI-F (5′-CGGCGGAATTCAGAAAACGAAACAAAGACGTAG-3′) and SpJC3EcoRI-F (5′-CAAAGGAATTCAGTTGAGCAAGCAGGGTCAGGC-3′), respectively. In all cases, primer XbaI-FlucR (5′-CCAAACTCATCAATGTATCTTATCATGTCTG-3′) was used as the reverse primer and plasmid dl sHBZ 5′UTR as the template. The amplicons were digested with EcoRI and XbaI and cloned into the intercistronic spacer of the dl sHBZ 5′UTR backbone previously digested with the same enzymes to remove the sHBZ 5′UTR and the FLuc ORF. The dl 1–45, dl 1–82 and dl 1–218 were obtained by PCR using the *Pfu* DNA polymerase with primers Sp45RncoI (5′-AAACGTCCATGGTCGAAAGTTCCACCCCTTTCC-3′) and Sp218RNcoI (5′-GTCTTCCATGGCCTCCTGAACTGCGTCCG-3′). The dl 1–82 construct was an unexpected product obtained using the primer Sp218RNcoI. Primer RlucNic1 was used as a forward primer and the dl sHBZ 5′UTR plasmid as the template. Amplicons were digested with XhoI and NcoI and cloned into the intercistronic space of the dl sHBZ 5′UTR backbone previously digested with XhoI and NcoI to remove the sHBZ 5′UTR. The monocistronic HCV IRES FLuc mRNA (HCV FLuc) was generated by PCR with the *Pfu* DNA polymerase using primers FV30 (5′-CCATATGTAATACGACTCACTATAGGGTTGGGGGCGACACTCC-3′) and ClaI-FlucR (5′-ATCGATTACACGGCGATCTTTCCG-3′) as described (22) and the dl HCV IRES plasmid as the template. All constructs used in this study were sequenced (Macrogen Corp., Rockville, MD, USA).

### Cell culture and cell extracts

COS-7 (ATTC CRL-1651), HeLa (ATTC CCL-2TM) and HEK 293T (ATCC CRL-11268) cells were grown as described in (15,23,24). Reagents for cell culture were purchased from HyClone, GE Healthcare Life Sciences (Logan, Utah, USA). To generate cell extracts, HeLa cells were grown to 60% confluence in DMEM (#SH30022) containing 10% fetal bovine serum (#SH30910), 1% penicilin–streptomicin (1000 U/ml) (#SV30010) and 1% fungizone (25 μg/ml) (#SV30078.01), at 37°C in a 5% CO_2_ atmosphere (nonsynchronized) or media supplemented with 400 ng/ml of nocodazole (#M14304, Sigma-Aldrich, St. Louis, MO) as previously described in (25).

### *In vitro* transcription

Plasmids were digested with XbaI except for the RLuc that was digested with Eco32I (EcoRV*) (*#ER0301), dl PV IRES with XhoI and Glo-FLuc with EcoRI. The RNAs were synthesized in a 50 μl *in vitro* transcription reaction for 2 h at 37°C using T7 RNA polymerase (#EP0111), 5 mM rNTPs, 1× Ribomax transcription buffer (80 mM 4-(2-hydroxyethyl)-1-piperazineethanesulfonic acid (HEPES)-KOH (pH 7.5), 24 mM MgCl_2_, 2 mM spermidine, 40 mM dithiothreitol (DTT)) and 20 U of RNAsin (#E00382). Then, RNA was treated with 2 U of Turbo DNase (#AM2238) for 30 min at 37°C. RNA was precipitated for 1 h at −20°C with 2.5 M LiCl, centrifuged at 16 000 g for 30 min at 4°C, washed with 70% ethanol and resuspended in 25 μl of nuclease-free water. RNA concentrations were determined spectrophotometrically using a Nanodrop 1000 (NanoDrop Technology, Wilmington, Delaware, USA), and RNA integrity was assessed by electrophoresis on denaturing formaldehyde agarose gel as detailed in (24). The capping of the mRNAs was carried out using the Vaccinia Capping System (#M2080; New England BioLabs, Ipswich, MA) according to the manufacturer’s specifications, and RNA polyadenylation was carried out using the Poly(A) tailing Kit using *E. coli* Poly(A) Polymerase (#M0276, New England BioLabs) according to the manufacturer’s specifications. Bicistronic mRNAs with a nonfunctional Cap (Acap) were synthesized in the presence of 3 mM A(5′)ppp(5′)G cap-analog (#S1406S; New England Biolabs).

### *In vitro* translation

*In vitro* translation reactions were carried out in nuclease-treated rabbit reticulocyte lysate (RRL; #L4960; Promega) as previously described (15). When HeLa cell extracts (S10) were used, the dl HIV-1 IRES bicistronic mRNA was preincubated for 10 min with 0.1 μg of cell extracts generated from G2/M arrested cells prior to the addition of the RRL translation mix as described previously (15,25). For *in vitro* translation reactions conducted in the presence of edeine, kindly provided by I. Brierley (University of Cambridge, UK), the protocol described in (15) was followed. Optimal salt concentrations were used to *in vitro* translate the monocistronic HCV-FLuc and dl HCV IRES (100 mM KCl and 0.75 mM MgOAc_2_), dl HIV-1 IRES (120 mM KOAc and 0.5 mM MgOAc_2_), dl PV IRES and dl HTLV-1 IRES (80 mM KOAc and 0.25 mM MgOAc_2_) mRNAs as previously described (13–15). Other monocistronic mRNAs were translated in RRL standard salt conditions (40 mM KOAc and 0.25 mM MgOAc_2_). All translation reaction mixtures were incubated at 30°C for 90 min. For ^35^S-methionine labeling, the RRL (Flexi^®^-RRL; #L4540; Promega) reactions were performed as described in (22), using 0.02 mM of amino acids lacking methionine and supplemented with 0.6 mCi*/*ml [^35^S]-methionine (PerkinElmer, Inc., Waltham, MA, USA). The translation reaction was stopped with 90 μl of protein loading buffer (10% sodium dodecyl sulphate, 50 mM Tris (pH 6.8), 10% glycerol, 100 mM DTT and 0.1% bromophenol blue) and 10 μl of the final reaction was resolved by sodium dodecyl sulphate-polyacrylamide gel electrophoresis (12%), and the labeled products were visualized and quantified using a Storm PhosphorImager (GE Healthcare) as specified in (22).

### DNA transfection

COS-7, HeLa or HEK 293T cells were seeded and transfected as indicated in (24). In the case of monocistronic vectors, 200 ng of the RLuc plasmid was co-transfected with 200 ng of musFluc, msFLuc, musFLucAAC or msFLucAAC. For transfection of bicistronic plasmids, cells were transfected with 200 ng of bicistronic DNA together with 50 ng of pcDNA 3.1-LacZ plasmid, encoding the β-galactosidase, which was used as a control for the transfection efficiency. At 24 h post-transfection, the culture medium was removed and the cells were harvested using Passive Lysis Buffer supplied with the DLR™ Assay System (#E194A; Promega) according to manufacturer’s protocols. For the poliovirus HRV 2A protease experiments COS-7 cells were cotransfected with 200 ng of the dl sHBZ 5′UTR plasmid and 500 ng of the HRV p2A-wt or HRV p2A-mut per well in a 24-well plate. Deferiprone (DFP) (CAS: 30652–11-0; #379409; Sigma-Aldrich) was used as described in (23). In these experiments 200 ng of dl sHBZ 5′UTR, dl HTLV-1 IRES or dl PV IRES were co-transfected with 50 ng of pcDNA3.1-LacZ in COS-7 cells. DFP was added 6 h post-transfection at 50 100 or 250 μM and 24 h post-transfection, the culture medium was removed and the cells were harvested.

### RNA transfection

COS-7 cells were seeded at 1.0 × 10^5^ cells per well in a 24-well culture plate and 0.4 pmol of an *in vitro* transcribed capped and polyadenylated RLuc RNA was co-transfected with 0.4 pmol of an *in vitro* transcribed capped and polyadenylated msFLuc or musFLuc RNA. The transfection was performed at 90% confluency using the Lipofectamine 2000 system (#11668019; ThermoFisher Scientific). Eight hours post-transfection, cells were harvested.

### siRNA–DNA co-transfection

RLuc RNA silencing was performed as described in (15,24). Twenty-four hours after transfection, the culture medium was removed and the cells were harvested. The eS25 knockdown protocole in COS-7 cells is described in detail in (24). During the second knockdown, 250 ng of dl sHBZ 5′UTR plasmid and 250 ng of pcDNA 3.1-LacZ plasmid were transfected together with the short interfering RNA (siRNA) as described in (24). Seventy-two hours post initial transfection, the cells were washed twice with 1× PBS and lysed with 1× PLB (Promega) for luciferase or β-galactosidase activity or with radioimmunoprecipitation assay buffer for western analysis (24).

### Luciferase assay

For eS25-siRNA experiments, luciferase assays were performed on 4 μl of lysate using the DLR™ Assay System (#E1960; Promega), and β-galactosidase activity was measured using 6 μl of lysate with the Galacto-Light Plus kit (ThermoFisher Scientific) according to the manufacturers’ protocols with a Lumat LB 9507 luminometer (Berthold Detection Systems GmbH). For all other luciferase assays 20 μl of cell lysate or 1 μl of RRL reaction was measured using a Sirius Single Tube Luminometer (Berthold). The activity of firefly luciferase (FLuc) and *Renilla* luciferase (RLuc) were measured using the DLR™ Assay System according to manufacturer’s instructions.

### Western analysis

Cells were lysed using 1× passive lysis buffer (supplied with the DLR™ Assay System) following the manufacturer’s instructions. The total protein content of all samples was determined by a Bradford assay using the Bio-Rad protein assay (Bio-Rad Laboratories, Inc., Hercules, CA, USA). In the HRV 2A protease experiments, 30 μg of total protein was resolved on 8% tris-glicine acrylamide gels and transferred to a nitrocellulose membrane (ThermoFisher Scientific). Membranes were blocked 1 h in TBS-T with 5% skim milk and later incubated with an anti-eIF4G antibody (sc-11373, H-300; Santa Cruz Biotechnology) at a 1:1000 dilution. For the GAPDH detection (loading control), an anti-GAPDH antibody (#MAS-15738; ThermoFisher Scientific) was used at a 1:5000 dilution. Either an anti-rabbit or anti-mouse IgG-horseradish peroxidase (HRP) (KPL, SeraCare, Milford, MA, USA) was used as secondary antibodies, at a 1:10 000 dilution. HRP westerns were visualized by enhanced luminescence using a chemiluminescence reaction using 4-hydroxycinnamic acid (#800237; Merck Millipore) and luminol (#09253; Fluka, Sigma-Aldrich). The procedure detailed in (24) was used to detect eS25 by western.

### RNA and DNA extraction, RT-PCR, RT-qPCR and PCR

RNA extraction was performed as described in (23). Cells were washed three times with 1× PBS (#SH30256; Hyclone) prior to the direct addition of TRIzol (#15596018; ThermoFisher Scientific), and RNA was purified according to the manufacturers’ protocol. Purified RNA was re-suspended in 25 μl of nuclease free water, DNase-treated for 30 min (#AM1907; ThermoFisher Scientific) and recovered according to the manufacturers’ instructions. RNAs were quantified by spectrophotometry using a Nanodrop 1000 (24). RNAs exhibited a ratio A260/A280 over 1.8 and their quality was monitored by electrophoresis on denaturing agarose gels and by using a standard sensitivity RNA analysis kit (#DNF-471; Advance Analytical Technologies) following the manufacturer’s protocol on a Fragment Analyzer Automated CE System (Advance Analytical Technologies). RNA quality analysis was made using the PROSize Data Analysis software. Samples used in the assay had a RNA quality number equal or over 8. When needed, RNA samples were stored at −80°C. The RT-PCR assay was carried out using the SuperScript™ III One-Step RT-PCR System with Platinum^®^ Taq DNA polymerase kit (#52122; ThermoFisher Scientific) according to manufacturer’s protocol, 5 ng of total RNA, and the primers Pforluc and p2anti as described in (15,26). The No-RT/PCR reaction was carried using the same amount of total RNA. Total DNA was extracted from COS-7 cells as described in (23), using the E.Z.N.A SQ Blood DNA Kit II (#D0714; Omega Bio-Tek, Inc, GA, USA) according to the manufacturers’ protocols, and total DNA (50 ng) was used in each PCR reaction with primers Pforluc and p2anti and GoTaq^®^ Green Master mix (#M7122; Promega), according to the manufacturers’ protocol, as described in (26). The RT-qPCR experiments were carried out in an optical tube 8× strip (#401428; Agilent Technologies, Santa Clara, CA, USA) using the Brilliant II SYBR Green qRT-PCR one Step Master Mix (#600835; Agilent Technologies), and 5 ng of total RNA per reaction and 10 μM of each primer in a final volume of 15 μl as detailed in (26). The amplification program consists of 30 min of reverse transcription at 50°C, 10 min of initial denaturing at 95°C and followed by 35 cycles (20 s denaturation step at 95°C, 20 s of annealing at 60°C and 30 s of extension at 72°C) of complementary DNA cDNA amplification. RLuc RNA was detected with Renilla sense (5′-AGGTGAAGTTCGTCGTCCAACATTATC-3′) and Renilla antisense (5′-GAAACTTCT TGGCACCTTCAACAATAGC-3′) set of primers (amplicon size: 193 bp; *R*^2^ = 0.9962; slope: −3.34; intercept: 36.76) and FLuc RNA was detected with firefly sense (5′-ACTTCGAAATGTCCGTTCGG-3′) and firefly antisense (5′-GCAACTCCGATAAATAACGCG-3′) primers (amplicon size: 135 bp; R^2^ = 0.9961; slope: -3.32; intercept: 36.62), as previously described (23,26,27). Ribosomal 18S rRNA, used as a reference gene, was detected with 18S sense (5′-GTGGAGCGATTTGTCTGGTT-3′) and 18S antisense (5′-CGCTGAGCCAGTCAGTGTAG-3′) primers (amplicon size: 200 bp; R^2^ = 0.993; slope: -3.375; intercept: 15.06). The 2^−ΔΔ*C*^_t_ method was used for the relative quantification of RNA (28). The reactions were carried out in a Stratagene Mx3000P thermal Cycler (Agilent Technologies) and the MXPro MX300P V4.10 Build 389, scheme 85 software was used to obtain the data. Further information required to comply with the Minimum Information for Publication of Quantitative Real-Time PCR Experiments (MIQE) guidelines checklist (29) not included in the above text are found in the Supplementary Materials and Methods section.

### RNA preparation, SHAPE Probing and RNA secondary structure modeling

The 5′UTR of the sHBZ mRNA was amplified from the msFLuc plasmids using T7pr (5′-TAATACGACTCACTATAGG-3′) as a forward primer and HBZ-SlFLuc (5′-AACCAGGGCGT ATCTCTTCATA-3′) as a reverse primer. RNA transcribed from the PCR fragment as described in (30) was precipitated with LiCl (2.5 M final concentration), washed with 70% EtOH and finally desalted by chromatography through a Sephadex G50 (GE Healthcare). RNA Selective 2′ hydroxyl acylation analysis by primer extension (SHAPE) analysis was conducted using 1-methyl-7-nitroisatoic anhydride (1M7) and N-methylisatoic anhydride (NMIA) as a modifying agents as previously described (31–34). RNA (6 pmol) was resuspended in 18 μl of water, denatured at 80°C for 2 min and cooled down at room temperature for 10 min in 40 mM HEPES (pH 7.5), 100 mM KCl, with or without 5 mM MgCl_2_. After a 10 min incubation at 37°C, RNAs were treated with 2 mM 1M7, or with 2 mM NMIA, or the equivalent volume of Dimethyl Sulfoxide (DMSO) and incubated for 30 min at 37°C. RNAs were cleaned by ethanol precipitation and pellets resuspended in 10 μl of water. Modifications were mapped by reverse transcription using the rHBZ-SlFLuc primer 5′fluorescently labeled (D2 or D4 WellRED, Sigma Aldrich) and M-MLV RNAse H^−^ reverse transcriptase (#M5301; Promega). RNAs were denatured for 3 min at 95°C with 1 μl of DMSO and cooled in ice for 3 min. Three microliters of primer was added and samples were incubated for 5 min at 65°C, 10 min at 35°C and finally cooled on ice. Reverse transcription was performed in several steps: 2 min at 35°C, 30 min at 42°C and 5 min at 55°C. The resulting cDNAs were separated by capillary electrophoresis (Beckman Coulter, Ceq8000). Data were analyzed using the software QuSHAPE (35). RNA probing was performed in triplicate for each RNA preparations. Significant SHAPE value differences in presence or absence of Mg^2+^ were defined as in (22). HTLV-1 sequences were recovered from the HTLV-1 database (http://htlv1db.bahia.fiocruz.br/) and manually aligned using bioEdit (http://www.mbio.ncsu.edu/BioEdit/page2.html). 1M7 and NMIA reactivity maps and sequence alignment were used to model RNA secondary structure using RNAalifold (36) following the workflow described in (37). RNA secondary structure was drawn using VaRNA (38).

### Sequence and statistical analysis

The statistical data analysis and graphics described in the text were done using the GraphPad v6.0c program (La Jolla, CA 92037, USA), while the BioEdit v7.0.9 (Ibis Biosciences, Carlsbad, CA 92008, USA) and the Serial cloner v2.6.1 were used for sequence alignments and analysis.

## RESULTS

### Synthesis of the HBZ isoforms are regulated at the level of translation initiation

**Figure 1.**
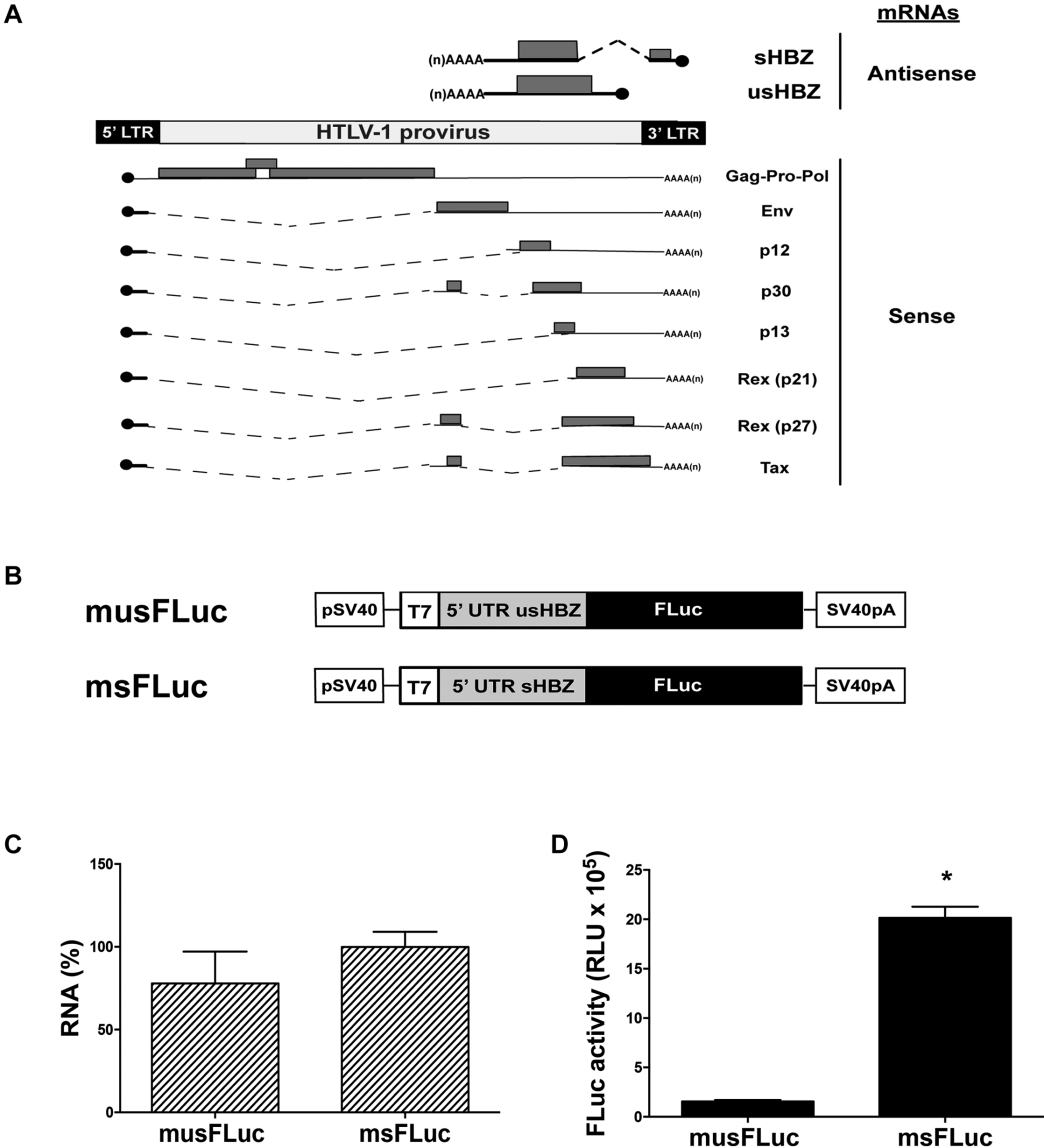
*In vitro* the 5′UTR of the *sHBZ* mRNA drives translation initiation more efficiently than the 5′UTR of the *usHBZ* mRNA. (**A**) Schematic representation of the HTLV-1 provirus and its mRNAs. The transcripts and proteins encoded by a complete HTLV-1 provirus are listed. Sense (below) and antisense (above) transcripts are depicted. For each mRNA, the 5′cap (black circle) and 3′poly(A) tail are shown. The introns that are spliced out for each transcript are indicated as dotted lines, while the ORFs are gray boxes and 5′ and 3′ UTRs are indicated by lines. (**B**) Schematic representation of the monocistronic DNA reporters used. The HBZ monocistronic FLuc DNA reporters contain the SV40 promoter and polyadenylation signal for their direct transfection in cells, as well as the T7 promoter to generate *in vitro* run-off transcripts. (**C** and **D**) The capped and polyadenylated musFLuc or msFLuc mRNAs were translated in RRL. Total RNA was extracted from RRL at the end of the reaction and quantified by RT-qPCR and presented as relative FLuc RNA levels normalized to the 18S rRNA; musFLuc was reported relative to msFLuc RNA, which was set to 100% (C). FLuc activity was determined and is presented as RLU (D). Values are the mean (+/− SEM) for three independent experiments (*n* = 3), each performed in duplicate. Statistical analysis was performed using a two-tailed *t*-test (*P < 0.05).

The HTLV-1 provirus allows both sense and antisense transcription (Figure 1A). The *sHBZ* and *usHBZ* antisense mRNAs differ only in their 5′UTR yet, they vary in translation efficiencies resulting in different levels of protein expressed (5,8). To determine whether the difference in translation could be attributed to their 5′UTRs (Supplementary Figure S1A), each was cloned upstream of a firefly luciferase (FLuc) reporter to generate plasmids msFLuc (sHBZ 5′UTR) and musFLuc (usHBZ 5′UTR) (Figure 1B). *In vitro* synthesized capped and polyadenylated monocistronic msFLuc and musFLuc mRNAs were translated in RRL and FLuc activity was measured. The results, expressed as relative luminescence units (RLU), show that despite similar mRNA levels in the RRL (Figure 1C), the msFLuc mRNA resulted in significantly higher (19-fold) FLuc activity than the musFLuc mRNA (Figure 1D). Next, the musFLuc and msFLuc RNA were then translated in RRL in the presence of ^35^S-methionine (^35^S-met), which demonstrated that the amount of synthesized protein correlated with the luciferase activity (Supplementary Figure S1B). Although other studies have indicated that sHBZ and usHBZ proteins have different half-lives (8), here we showed that differences in rates of synthesis also contributed to differences in protein expression levels. Since we have used reporters that encode for the exact same reporter protein, FLuc, we have eliminated effects on protein stability in our system. Therefore, our results showed that the sHBZ 5′UTR drove synthesis of the FLuc protein more efficiently than the usHBZ 5′UTR in RRL.

**Figure 2.**
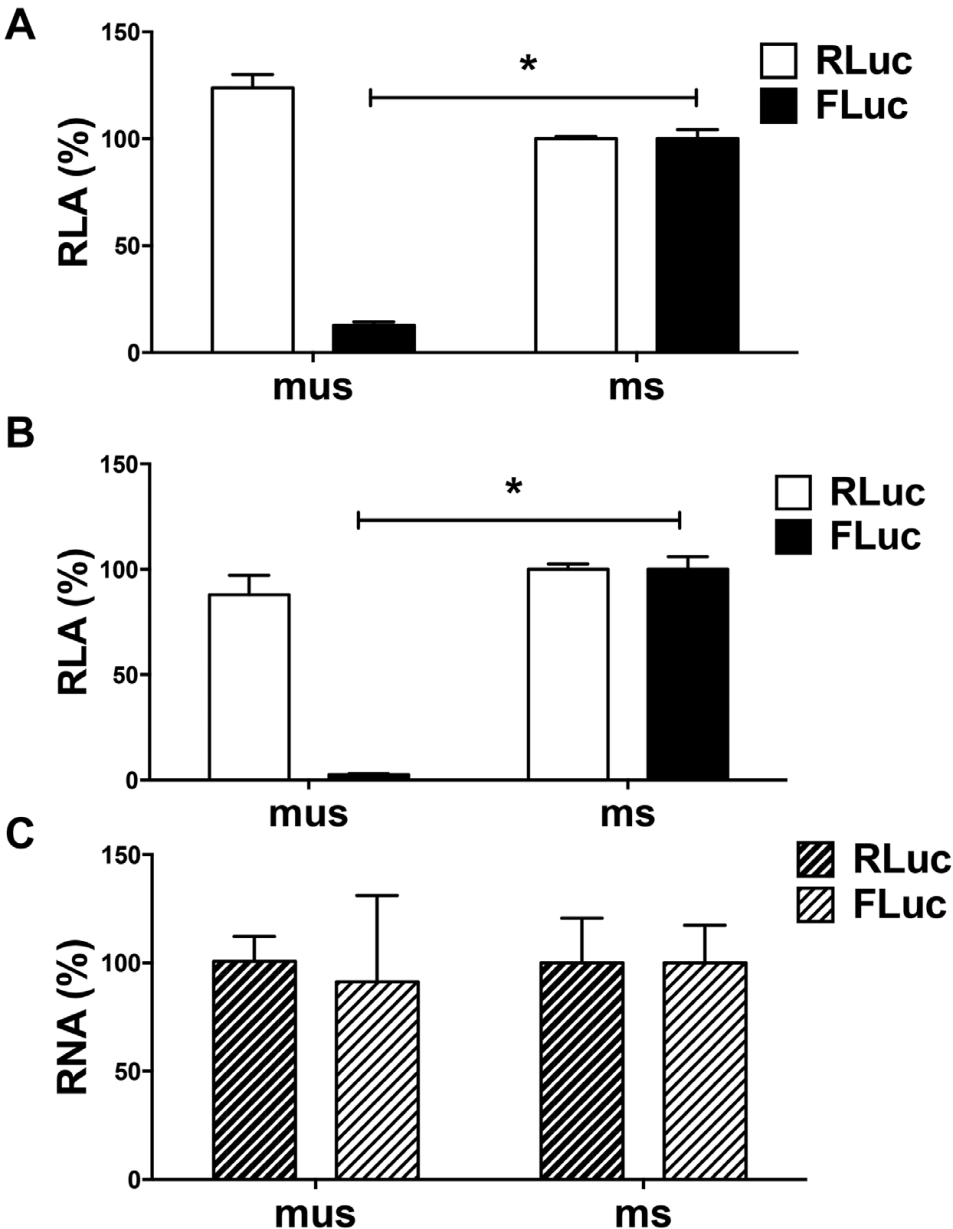
The sHBZ 5′UTR drives translation initiation more efficiently than the 5′UTR of the *usHBZ* mRNA in cell culture. (**A**) The msFLuc or musFLuc DNA plasmid was co-transfected into COS-7 cells together with a plasmid encoding RLuc, which was used to control for transfection efficiency. Luciferase activity was measured 8 h after transfection and reported as % RLA to msFLuc activity, which was set to 100%. Values shown are the mean (+/− SEM) for four independent experiments, each performed in duplicate. (**B** and **C**) *In vitro* transcribed capped and polyadenylated musFLuc or msFLuc mRNAs were co-transfected into COS-7 cells with a capped and polyadenylated RLuc mRNA. (B) Luciferase activities measured at 24 h post-transfection and are shown as RLA with the msHBZ RNA set to 100%. (C) RT-qPCR on total RNA extracted from cells was used to quantify the RLuc or FLuc RNA levels. The RNA was normalized to the 18S rRNA level and presented as relative to the msHBZ Fluc RNA, which was set to 100%. Values shown are the mean (+/− SEM) for three independent experiments, each performed in duplicate. Statistical analysis was performed using a two-tailed *t*-test (*P < 0.05).

To determine if the differences that we observed in translational efficiency in RRL also prevailed in cells, DNA reporters encoding msFLuc and musFLuc (Figure 1B) were transfected into COS-7 cells along with a plasmid encoding the *Renilla* luciferase (RLuc) reporter (16), which served as a control for transfection efficiency between samples. Data were expressed as relative luciferase activities (RLA). Similar to our *in vitro* findings, the FLuc activity driven from the sHBZ 5′UTR (set to 100%) was much higher (∼8-fold) than from the usHBZ 5′UTR (Figure 2A). Importantly, equivalent levels of RLuc activity suggested that variations in FLuc activity were not attributable to differences in transfection efficiency (Figure 2A). Comparable results were also obtained from transfecting *in vitro* transcribed capped and polyadenylated musFLuc or msFLuc mRNAs into COS-7 cells together with a capped and polyadenylated RLuc mRNA (Figure 2B). The levels of *in vitro* transcribed RNA recovered from transfected cells were equivalent for both the musFLuc and msFLuc transfected COS-7 cells (Figure 2C). Together, these findings indicate that the 5′UTR of the *sHBZ* mRNA promotes more efficient translation initiation than the 5′UTR of the *usHBZ* mRNA *in vitro* and *in vivo*.

### Impact of upstream initiation codons on translation initiation driven by the 5′UTR of the *usHBZ* and *sHBZ* mRNA

**Figure 3.**
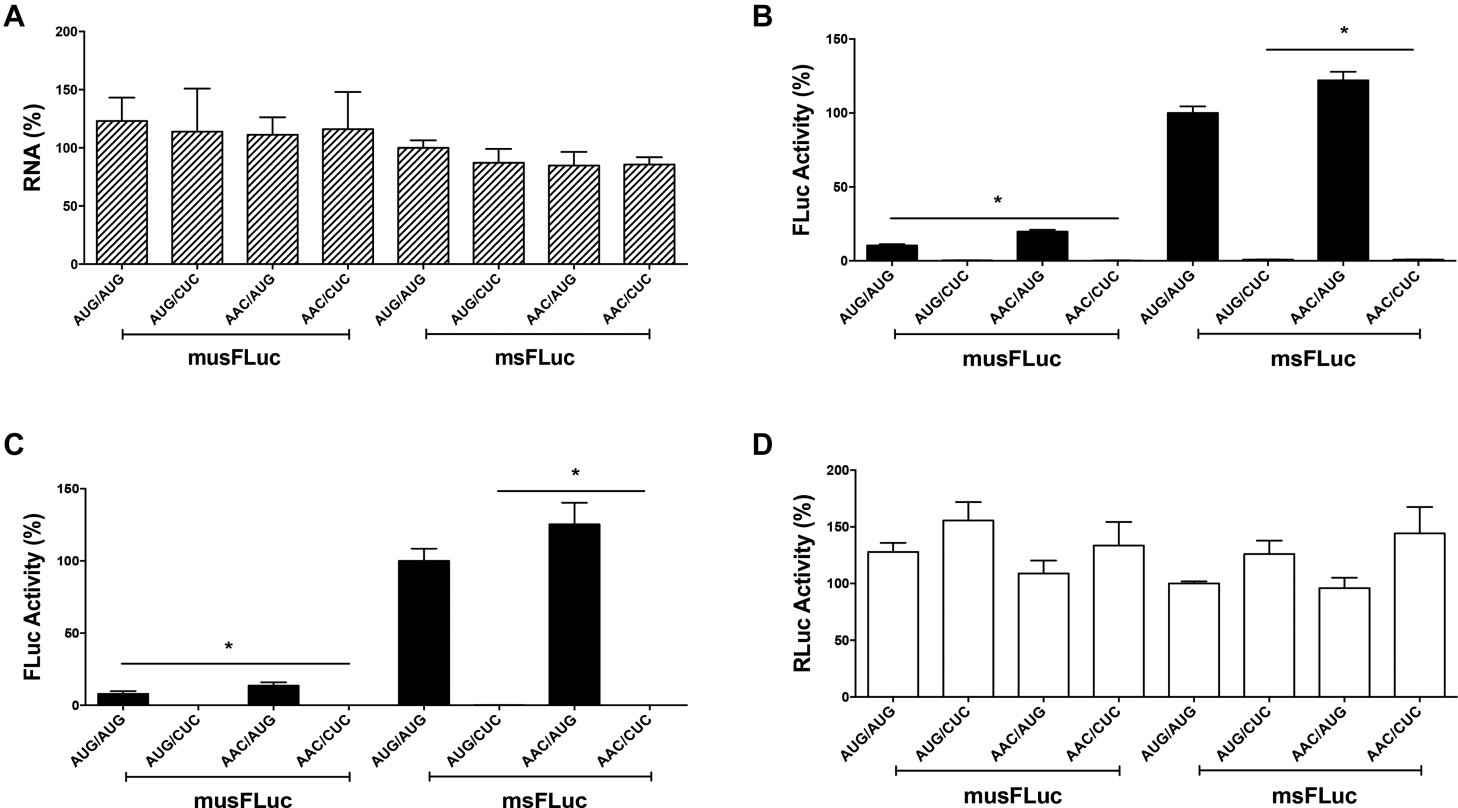
Upstream initiation codons (uAUGs) have little effect on translation initiation driven by the 5′UTR of the *sHBZ* or *usHBZ* mRNAs. The upstream AUGs (uAUGs) in the 5′UTR of the *usHBZ* and *sHBZ* mRNAs (Supplementary Figure S1A) were mutated to AAC. When indicated the initiation codon (iAUG) was mutated to CUC. (**A** and **B**) The capped and polyadenylated msFLuc (sHBZ 5′UTR uAUG/iAUG), msFLuc CUC (sHBZ 5′UTR uAUG/CUC), musFLuc (usHBZ 5′UTR uAUG/iAUG), musFLuc CUC (usHBZ 5′UTR uAUG/CUC), msFLucAAC (sHBZ 5′UTR AAC/iAUG), msFLuc AAC/CUC (sHBZ 5′UTR AAC/CUC), musFLucAAC (usHBZ 5′UTR AAC/iAUG) or musFLucAAC/CUC (usHBZ 5′UTR AAC/CUC) mRNAs with the uAUG or without (AAC), and with the iAUG or without (CUC) were translated in RRL. (A) Total RNA was extracted from the end of the reaction and quantified by RT-qPCR and presented as relative FLuc RNA levels normalized to the 18S rRNA; musFLuc was reported relative to msFLuc RNA, which was set to 100%. (B) FLuc activity was determined and is reported as (%) FLuc relative to that obtained from the msFLuc (sHBZ 5′UTR uAUG/iAUG) plasmid, which was set to 100%. Values are the mean (+/− SEM) for four independent experiments (*n* = 4), each performed in duplicate. Statistical analysis was performed using a two-tailed *t*-test (*P < 0.05). (**C** and **D**) The sHBZ or usHBZ DNA plasmids encoding for monocistronic mRNAs, with the uAUG or without (AAC), or with the iAUG or without (CUC) were transfected into COS-7 cells together with the transfection control RLuc plasmid. (C) Luciferase activities were measured 24 h post-transfection and FLuc activities are presented as (%) FLuc activity relative to the FLuc activity obtained from the msFLuc (sHBZ 5′UTR uAUG/iAUG) plasmid, which was set to 100%. (D) RLuc activities from the transfection control, RLuc plasmid are presented relative to that obtained when the msFLuc plasmid was co-transfected (sHBZ 5′UTR uAUG/iAUG), which was set to 100%. Values shown are the mean (+/− SEM) for at least three independent experiments, each performed in duplicate. Statistical analysis was performed by an ANOVA test followed by a Dunnett’s multiple test comparison (*P < 0.05).

**Figure 4.**
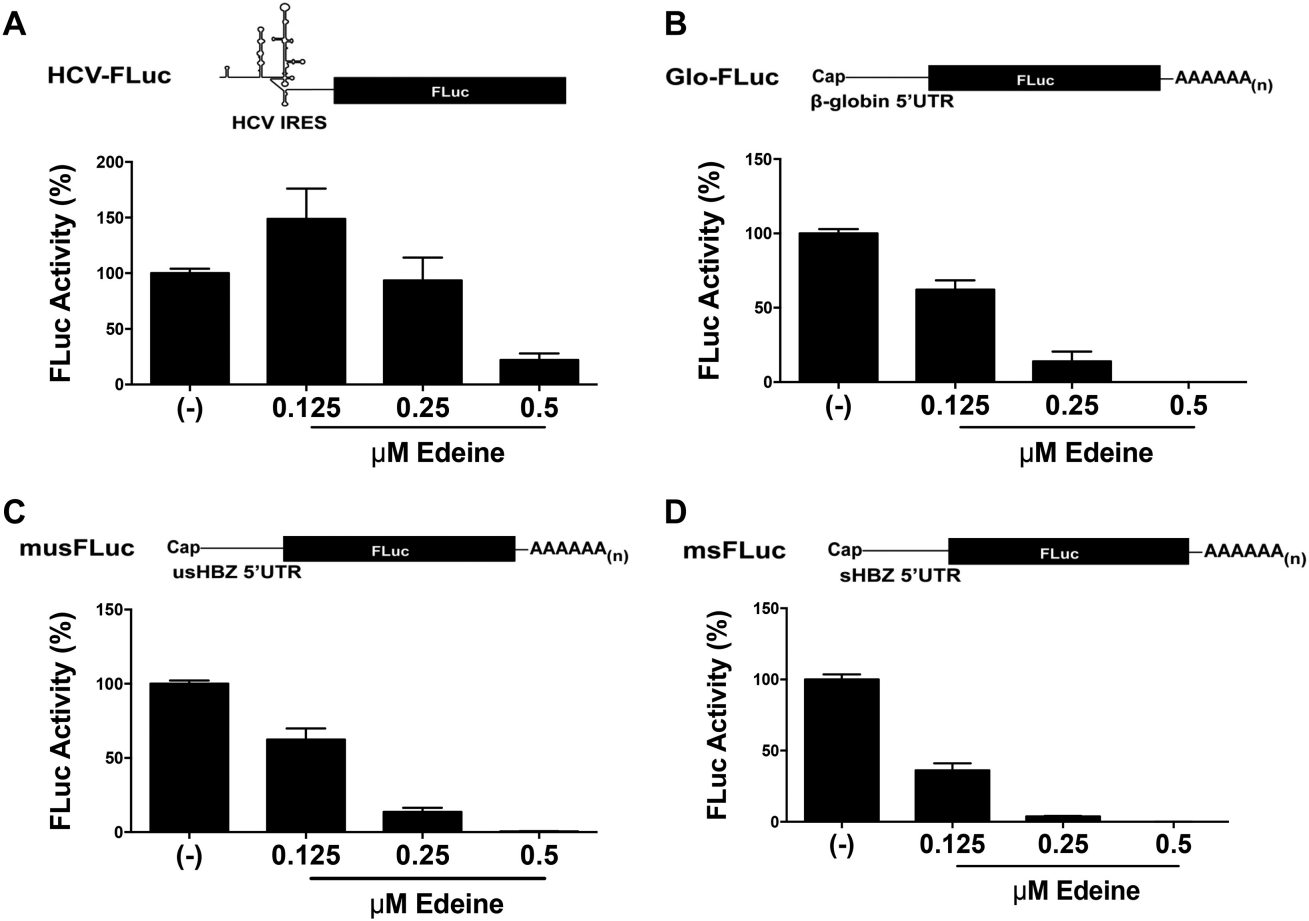
The usHBZ and sHBZ 5′UTRs rely on 40S scanning for initiation codon recognition. The *in vitro* transcribed monocistroinic HCV-FLuc (**A**), capped and polyadenylated Glo-FLuc, which harbors the 5′UTR of scanning-dependent globin mRNA (**B**), capped and polya.denylated musFLuc (**C**), or capped and polyadenylated msFLuc (**D**). mRNAs were *in vitro* translated in RRL in the absence (−) or the presence of increasing concentrations of edeine (0.125, 0.25 or 0.5 μM) and the FLuc activity was measured after a 90 min reaction as indicated in the ‘Materials and Methods’ section. The FLuc activity is reported as % FLuc relative to the FLuc activity in the absence (−) of drug set to 100%. Values are the mean (+/− SEM) for three independent experiments, each performed in duplicate.

The presence of upstream AUGs (uAUGs) codons upstream of the authentic start codon is known to repress initiation at the downstream start codon (9). Given the presence of uAUGs within the *sHBZ* and *usHBZ* 5′UTRs, we sought to determine if they impacted on initiation from the authentic HBZ start codon (iAUG). All uAUGs present within the *usHBZ* (four) and *sHBZ* (one) 5′UTRs (Supplementary Figure S1A) were mutated to AAC keeping the iAUG intact (msFLuc AAC/AUG and musFLuc AAC/AUG). In addition, the iAUG was mutated to CUC (AUG/CUC) in the musFLuc and msFLuc background and in the context of the AAC mutant constructs (AAC/CUC). *In vitro* synthesized capped and polyadenylated mRNAs were translated in RRL and FLuc activity was measured. The results show that despite recovering comparable amounts of mRNA from the RRL reaction (Figure 3A), the removal of uAUGs from the usHBZ 5′UTR (AAC/AUG) increased (1.9-fold) FLuc expression (Figure 3B). Even though the increase in FLuc translation, exhibited by the (AAC/AUG) musFLuc mRNA, was significant when compared to the (AUG/AUG) msFLuc mRNA, it remained marginal when compared to the FLuc activity obtained from the (AUG/AUG) msFLuc RNA. Removal of the uAUG from the sHBZ 5′UTR (AAC/AUG) induced a non-significant increase (1.2-fold) in FLuc expression (Figure 3B). As expected, no FLuc activity was detected when the iAUG was replaced by CUC, indicating that *in vitro* translation initiation leading to the synthesis of a functional FLuc protein occurs only from the expected initiation codon (Figure 3B). Similar findings were obtained in COS-7 cells when DNA reporters were transfected together with the RLuc expressing plasmid (used to control for transfection efficiency). Briefly, removal of uAUGs from the usHBZ 5′UTR (musFLucAAC/AUG) increased translation (1.7-fold) but never to levels obtained for the sHBZ 5′UTR (msFLucAUG/AUG) containing mRNA (7.5-fold higher) (Figure 3C). Removal of the uAUG from the sHBZ 5′UTR (AAC/AUG) marginally increased expression of FLuc (1.2-fold) (Figure 3C). Confirming *in vitro* observations that no FLuc activity was detected when the iAUG was replaced by CUC. The presence of RLuc activity (Figure 3D) confirmed that in all cases DNA had been transfected and indicated that differences in the FLuc activities between the different reporters cannot be attributed to the differences in transfection efficiencies. If the four uAUGs were responsible for reducing the translation of the usFLuc reporter then changing them should have completely relieved this repression; however, since this was not the case, this suggested that other factors besides the uAUGs were responsible for the differences in translation initiation of the *usHBZ* and *sHBZ* 5′UTR reporters. Thus, we wondered whether initiation codon recognition required ribosome scanning. To explore this possibility, *in vitro* translation assays (RRL) were performed in the presence of edeine, a drug interferes with initiation codon (AUG) recognition by the scanning 40S-eIF2-GTP/Met-tRNAi complexes (39,40). At low concentrations edeine inhibits start codon recognition during 40S scanning, but at higher concentrations it inhibits elongation (41–43). Therefore, we established the conditions that inhibited 40S recognition of the start codon during scanning using an mRNA that is dependent on scanning, Glo-FLuc mRNA (5′UTR of globin), and an mRNA that initiates independent of scanning, HCV-FLuc (hepatitis C virus IRES). Translation reactions were conducted in the absence or the presence of increasing concentrations of edeine. The HCV IRES promoted translation of FLuc at concentrations up to 0.25 μM of edeine but was inhibited at higher concentrations (Figure 4A), consistent with previous findings (41). The reporter activity for the Glo-FLuc mRNA decreased in a concentration-dependent manner even beginning at the lowest dose (0.125 μM) of edeine (Figure 4B). These controls establish that at concentrations between 0.125 and 0.25 μM of edeine codon recognition by a scanning 40S ribosomal subunit is inhibited, but not elongation. Next, we evaluated the effect of edeine on translation initiation driven by the usHBZ and sHBZ 5′UTRs. FLuc reporter activity for both the musFLuc (Figure 4C) and msFLuc (Figure 4D), mRNAs was reduced at the lowest concentration of edeine (0.125 μM), which suggests that 40S scanning was required to recognize the HBZ initiation codon for both usHBZ and sHBZ reporter mRNAs.

### The sHBZ 5′UTR can initiate translation in the context of a bicistronic mRNA, while the usHBZ 5′UTR cannot

**Figure 5.**
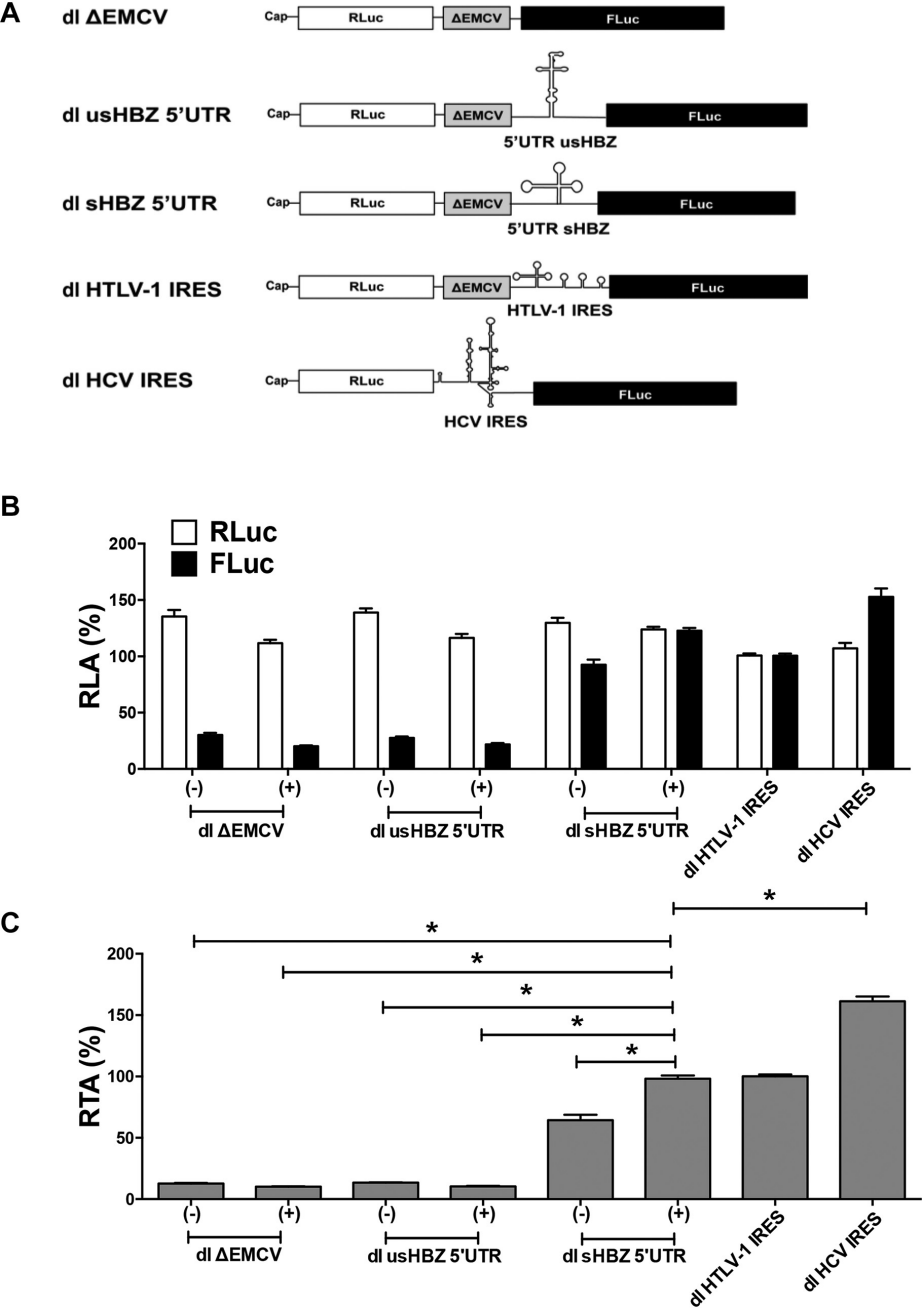
The sHBZ 5′UTR can initiate translation in the context of a bicistronic mRNA *in vitro*. (**A**) Schematic representation of the dual luciferase (dl) RNAs used. RNAs were *in vitro* transcribed with a 5′cap structure. The first cistron, RLuc is translated cap-dependently while translation of the second cistron, FLuc is dependent on the presence of an IRES in the intragenic region. The ΔEMCV element serves as a highly structured region and is expected to prevent readthrough or reinitiation following translation of RLuc. (**B** and **C**) *In vitro* transcribed capped dl usHBZ 5′UTR, dl sHBZ 5′UTR or dl ΔEMCV RNAs were translated in RRL (−) or in salt-optimized RRL (+) (Supplementary Figure S2). The control dl HTLV-1 IRES and dl HCV IRES dl RNAs RRL optimized for each IRES activity as described in ‘Materials and Methods’ section. RLA (B) or relative translation activity (RTA) (C) are reported relative to the capped dl HTLV-1 IRES activity, which was set to 100%. Values are the mean (+/− SEM) for at least four independent experiments, each performed in duplicate. RTA corresponds to the FLuc/RLuc ratio that is used as an index of IRES activity. Statistical analysis was performed by an ANOVA test followed by a Dunnett’s multiple test comparison (*P < 0.05).

Since several retroviral mRNAs, including the 5′UTR of the full-length HTLV-1 mRNA have IRES activity (15,44), we next sought to determine whether translation initiation driven from the usHBZ and sHBZ 5′UTRs could occur by internal initiation. For this, the 5′UTRs were inserted into a dual luciferase (dl) reporter containing an upstream *RLuc* coding region and a downstream *FLuc* coding region (Figure 5A). Following previously reported strategies (13,15), a highly structured defective EMCV IRES sequence (ΔEMCV) was inserted upstream of the usHBZ and sHBZ 5′UTRs (Figure 5A). In this context, the RLuc reporter is translated by a cap-dependent mechanism, while FLuc expression is dependent on whether the usHBZ or sHBZ 5′UTRs can initiate translation internally. The HCV and HTLV-1 IRESs inserted into bicistronic reporter vectors (14,15) and served as positive controls for IRES activity, while the dl ΔEMCV reporter functioned as a negative control (Figure 5A). Capped bicistronic dl usHBZ 5′UTR, dl sHBZ 5′UTR or dl ΔEMCV RNAs were synthesized and translated in RRL or salt-optimized RRL (Supplementary Figure S2; 80 mM KOAc and 0.25 mM MgOAc_2_). The control dl HTLV-1 IRES and dl HCV IRES RNAs were translated in salt-optimized RRL as previously reported (14,15). The cap-dependent reporter RLuc was expressed to similar levels from all RNAs (Figure 5B). The FLuc activity obtained from the dl ΔEMCV mRNA was considered background as it represents the leakiness in luciferase expression of the bicistronic system. The sHBZ 5′UTR promoted FLuc translation in both non-optimized and salt-optimized RRL (Figure 5B). As expected (Supplementary Figure S2), expression of FLuc driven by the sHBZ 5′UTR was higher when the RRL was salt optimized (Figure 5B). The FLuc activity from the usHBZ 5′UTR was similar to the negative control, ΔEMCV (Figure 5B) indicating that it was unable to drive FLuc translation, thus internal initiation. Analysis of the FLuc/RLuc ratio (relative translational activity, RTA), index of IRES activity, better illustrates the significantly higher (*P* < 0.05) sHBZ 5′UTR activity compared to the ΔEMCV background (4.5- to 7.5-fold), while activity driven by the usHBZ 5′UTR was at background levels (Figure 5C). This suggests that the sHBZ but not the usHBZ 5′UTR is capable of promoting internal initiation in RRL.

To determine if the ΔEMCV sequence in combination with the sHBZ 5′UTR could give rise to an artificial IRES element, capped bicistronic mRNAs containing the ΔEMCV sequences (dl sHBZ 5′UTR) or not (dl ΔΔEMCV sHBZ 5′UTR) were translated in RRL (Supplementary Figure S3). The sHBZ 5′UTR by itself could drive cap-independent translation initiation in the context of a bicistronic mRNA (Supplementary Figure S3). Since translation from the second cistron increased (2-fold) in the absence of the ΔEMCV element, this indicated that the ΔEMCV element did not contribute to IRES-mediated translation initiation from the sHBZ 5′UTR in the context of the dl sHBZ 5′UTR RNA *in vitro* (Supplementary Figure S3). To further confirm that both cistrons were independently translated, we blocked cap-dependent translation by capping with an A-cap (ApppG), cap-analog that is not recognized by the cap-binding protein, eIF4E (45). RRL was programmed with *in vitro* transcribed dl ΔEMCV, dl HTLV-1 IRES and dl sHBZ 5′UTR RNA harboring either a functional 5′^m7^GpppG (cap) or an Acap. Translation of the cap-dependent cistron (RLuc), but not sHBZ 5′UTR-mediated FLuc translation, was reduced when the mRNA had an A-cap compared to when it had a 5′^m7^GpppG cap (Supplementary Figure S4), confirming that in RRL translation of the second cistron is translated independent of the first cistron. Together these findings suggest that the sHBZ 5′UTR has IRES activity *in vitro*.

### The sHBZ 5′UTR mediates cap-independent translation initiation in cells

**Figure 6.**
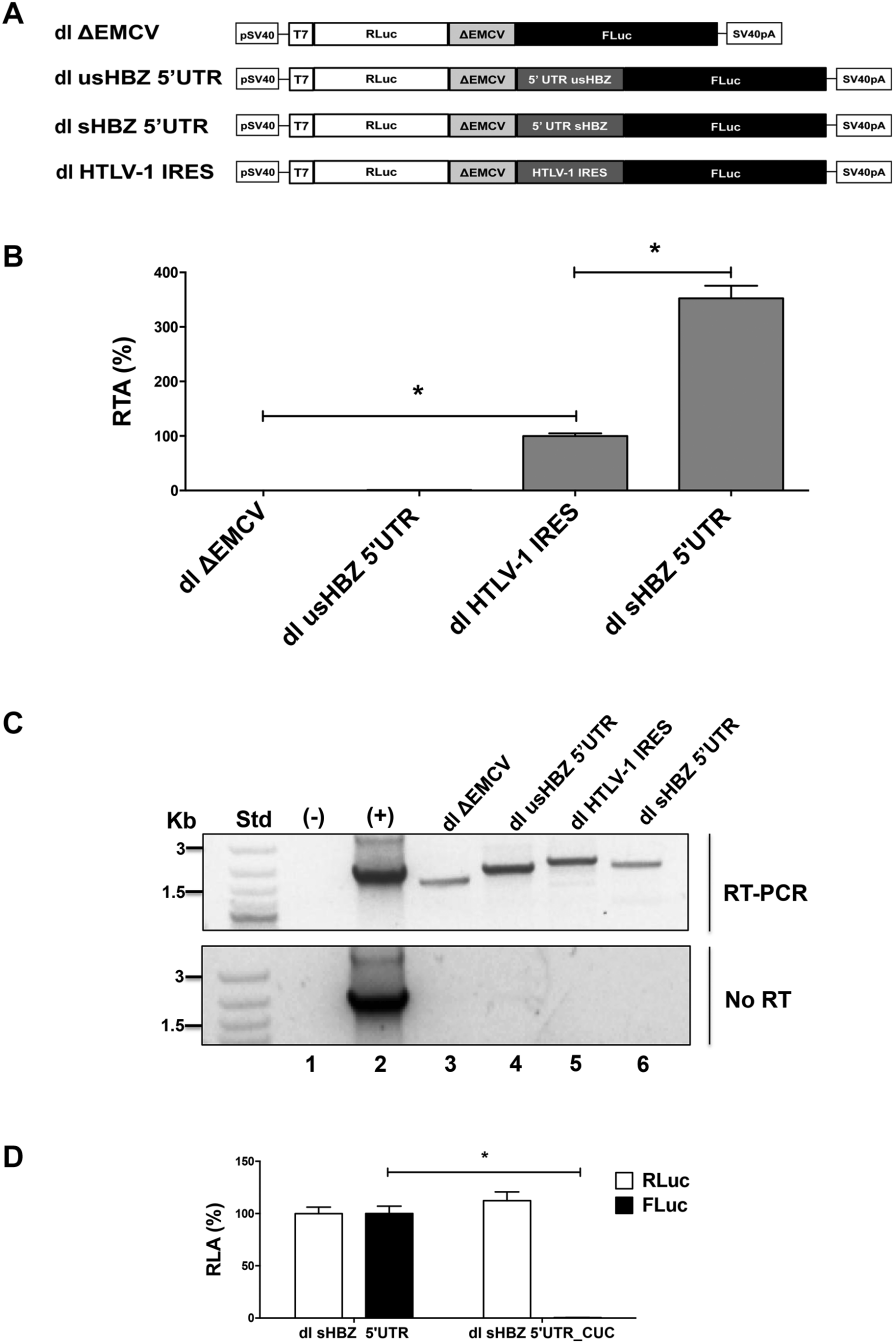
The sHBZ 5′UTR mediates cap-independent translation initiation in cells. (**A**) Schematic representation of the dual luciferase (dl) vectors used in this assay. All vectors harbored the SV40 promoter and poly(A) signal. (**B** and **C**) The dl ΔEMCV, dl HTLV-1 IRES, dl usHBZ 5′UTR or dl sHBZ 5′UTR reporter plasmids were co-transfected into COS-7 cells with the transfection control pcDNA3.1 *lac*Z plasmid that expresses β-galactosidase from a cap-dependent transcript. Total RNA and protein lysates were harvested from cells 24 h after transfection. (B) RLuc and FLuc activities were determined and normalized to the β-galactosidase activity. Values are expressed as RTA (%) relative to the dl HTLV-1 IRES, which was set to 100%. Values are the mean (+/− SEM) for four independent experiments, each performed in duplicate. Statistical analysis was performed by an ANOVA test followed by a Dunnett’s multiple test comparison (*P < 0.05). (C) RT-PCR was performed on total RNA extracted from transfected cells to detect the full-length dl mRNA using primers that hybridize to the RLuc and the FLuc sequences (top panel). The amplification reaction in absence of DNA or RNA was used as a negative control (−), while plasmid DNA was used as a positive control (+). As an additional control for DNA contamination, the one-step RT-PCR reaction was also conducted in the absence of reverse transcriptase (no RT; lower panel). (**D**) The dl sHBZ 5′UTR or dl sHBZ 5′UTR_CUC reporter plasmids were co-transfected into COS-7 cells with the transfection control pcDNA3.1 *lac*Z plasmid that expresses β-galactosidase from a cap-dependent transcript. In the dl sHBZ 5′UTR_CUC plasmid, the FLuc iAUG has been replaced by CUC. RLuc and FLuc activities were determined and normalized to the β-galactosidase activity. Values are expressed as RLA (%) relative to the dl sHBZ 5′UTR, which was set to 100%. Values shown are the mean (+/− SEM) for three independent experiments, each performed in duplicate. Statistical analysis was performed using a two-tailed *t*-test (*P < 0.05).

To investigate whether the usHBZ or sHBZ 5′UTRs exhibit IRES activity in cells, the dl usHBZ 5′UTR, dl sHBZ 5′UTR, dl HTLV-1 IRES or dl ΔEMCV plasmids (Figure 6A) were transfected into COS-7 cells. Twenty-four hours post-transfection total protein was extracted from the COS-7 cells and RLuc and FLuc activity was determined (Figure 6B). In agreement with the RRL data, the 5′UTR of the *usHBZ* mRNA had no IRES activity, while the 5′UTR of the *sHBZ* mRNA promoted FLuc translation in COS-7 cells (Figure 6B). In COS-7 cells, the 5′UTR of *sHBZ* mRNA exhibited significantly (*P* < 0.05) more translational activity (3.5-fold) than the HTVL-1 IRES (Figure 6B), used as the IRES control. Similar observations were made when plasmids were transfected in HeLa (Supplementary Figure S5A) and HEK 293T (Supplementary Figure S5B) cells. RT-PCR of the total RNA from the transfected COS-7 cells demonstrated that all of the transfected reporter plasmids generated a full-length bicistronic mRNAs (Figure 6C; top panel). Even though these results were not quantitative nor did they rule out the presence of other RNA species, they confirmed the expression of the full-length bicistronic mRNA within cells. No amplicon was observed when the PCR reaction was conducted without reverse transcription (Figure 6C; lower panel) confirming the absence of DNA contamination in the RNA preparation. When the HBZ initiation codon in the dl sHBZ 5′UTR construct was replaced by CUC (dl sHBZ 5′UTR_CUC) only RLuc activity was detected in transfected COS-7 cells indicating that synthesis of the functional FLuc protein from the bicistronic mRNA only starts at the expected initiation codon (Figure 6D).

**Figure 7.**
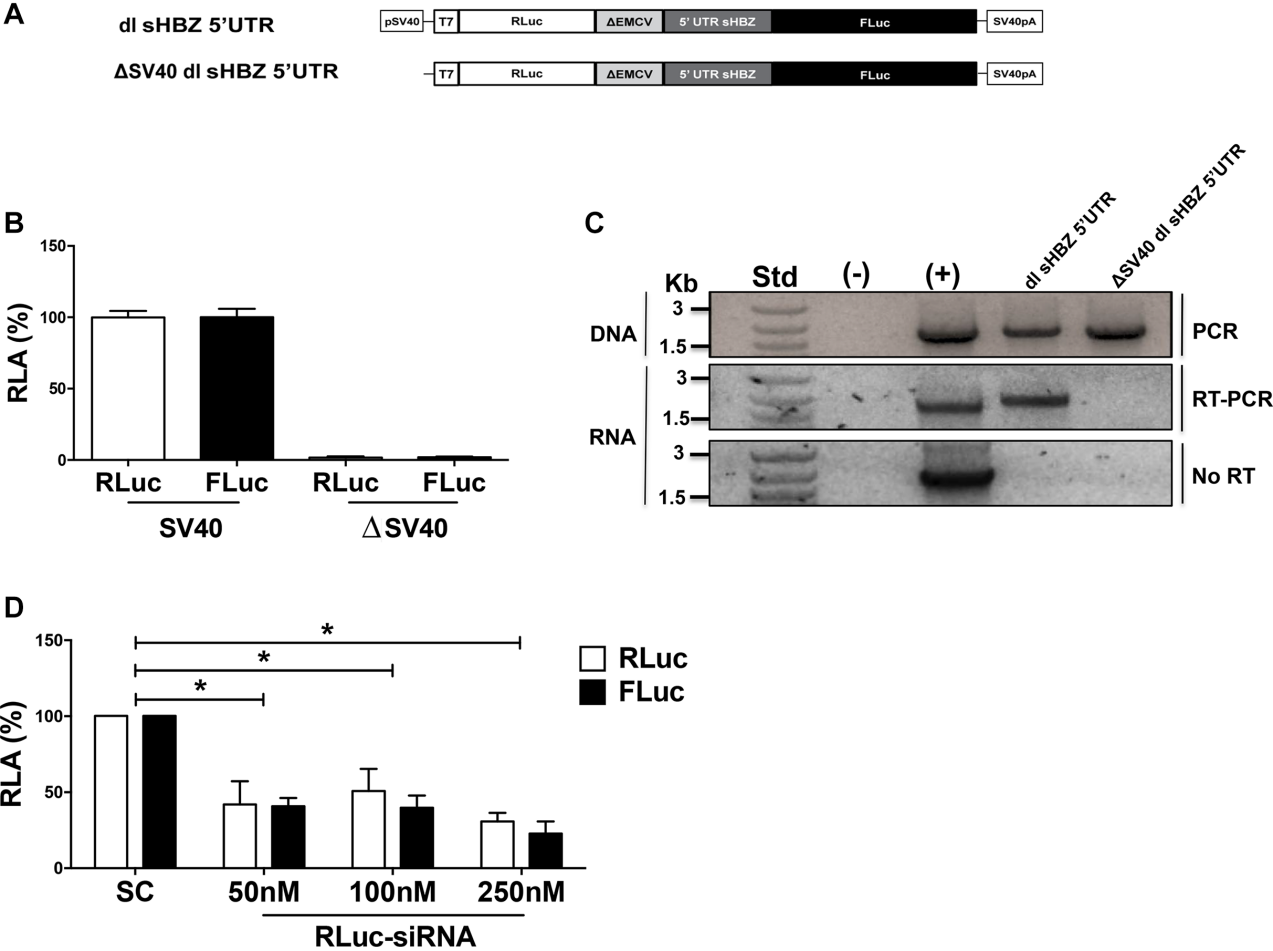
Cryptic promoter or splicing activity does not account for the FLuc activity. (**A**) Schematic representation of the bicistronic constructs. The SV40 promoter from dl sHBZ 5′UTR was removed to generate the promoterless (ΔSV40) vector. SV40pA represents the SV40 polyadenylation signal. COS-7 cells were co-transfected with plasmids containing the depicted reporters with the pcDNA3.1 *lac*Z plasmid. Total DNA, RNA and proteins were extracted from cells 24 h after transfection. (**B**) RLuc and FLuc activities were measured and normalized to the β-galactosidase activity. Values are expressed as RLA (%) relative to the RLuc and FLuc activities obtained from the dl sHBZ 5′UTR (with the SV40 promoter) plasmid, set to 100%. Values are the mean (+/− SEM) for three independent experiments, each performed in duplicate. (**C**) PCR was performed on total DNA isolated from the cells (top panel), while an RT-PCR was performed on total RNA. Primers that hybridized to the RLuc and the FLuc sequences were designed to detect the full-length bicistronic mRNA (middle panel). As an additional control, the one-step RT-PCR reaction was conducted in the absence of reverse transcriptase (No RT-PCR, bottom panel). The amplification reaction in absence of DNA or RNA was used as a negative control (−), while plasmid DNA was used as a positive control (+). (**D**) Approximately 50, 100 or 250 nM of scrambled control siRNA, or an siRNA that targeted the *Renilla* luciferase ORF, was co-transfected with the dl sHBZ 5′UTR plasmid. RLuc and FLuc activities were measured 24 h post-transfection and expressed relative to the scrambled control siRNA set to 100% (RLA). Values shown are the mean (+/− SEM) for three independent experiments, each performed in duplicate. Statistical analysis was performed by an ANOVA test followed by a Dunnett’s multiple test comparison (*P < 0.05).

To exclude the existence of possible cryptic promoter sequences within the dl-plasmid that give rise to functional monocistronic capped-FLuc mRNAs, the SV40 promoter was removed from the dl sHBZ 5′UTR plasmid generating the ΔSV40 dl sHBZ 5′UTR vector (Figure 7A). The dl sHBZ 5′UTR or the promoterless ΔSV40 dl sHBZ 5′UTR plasmids were transfected into COS-7 cells and 24 h later DNA, RNA and protein were extracted. In the absence of the SV40 promoter, RLuc and FLuc activities were not above background (Figure 7B). RT-PCR of the total RNA, confirmed that the bicistronic (RLuc-FLuc) RNA was found only in cells transfected with the plasmids containing an SV40 promoter (Figure 7C; middle panel). PCR analysis confirmed that the cells were transfected with the dl vectors (Figure 7C; top panel). Importantly, the RT-PCR product was dependent on reverse transcription (Figure 7C; No-RT, lower panel), demonstrating that the amplified product was from RNA and not from contaminating DNA. Taken together, these findings demonstrate that the FLuc activity cannot be attributed to the presence of a cryptic promoter within the dl sHBZ 5′UTR plasmid. Similar results were obtained when the SV40 and ΔSV40 vectors were transfected into HeLa (Supplementary Figure S5C) or HEK 293T (Supplementary Figure S5D) cells. However, these cell lines exhibited a residual promoter activity of 12% (HeLa) and 14% (HEK 293T) suggesting that in these cell lines most, but not all, FLuc activity can be attributed to an IRES. Based on these findings, we decided not to use HeLa or HEK 293T cell lines in future experiments.

The possibility remained that an alternative splicing event could generate a monocistronic cap-dependent mRNA that encoded for a functional FLuc protein. To explore this possibility, we targeted the *Renilla* ORF with a siRNA (15,24). Increasing concentrations of *Renilla* siRNA (RLuc-siRNA) or a scrambled control (SC) siRNAs were co-transfected with the dl sHBZ 5′UTR in COS-7 cells and the RLuc and FLuc activities were determined. In the presence of the siRNA targeting *Renilla*, both RLuc and FLuc activities were reduced (Figure 7D), strongly suggesting that both reporter proteins are synthesized from the same transcript.

### The HBZ 5′UTR directs translation in cells when eIF4G is cleaved by the HRV 2A protease

**Figure 8.**
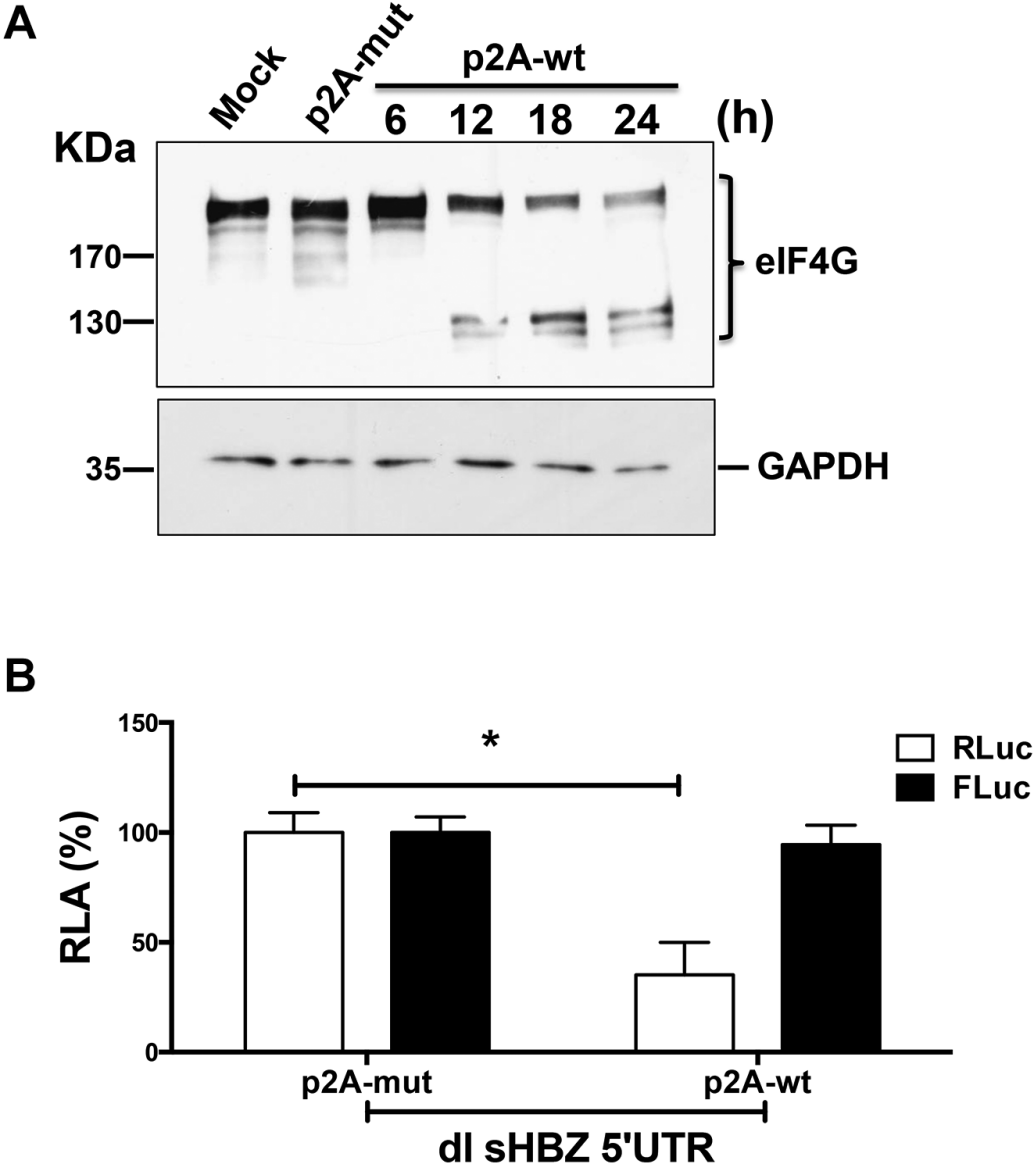
Translation promoted by the sHBZ 5′UTR is resistant to the proteolytic cleavage of eIF4G by the HRV 2A protease. (**A**) COS-7 cells were transfected with plasmids expressing the wt (p2A-wt) or mutant (p2A-mut) HRV 2A protease. Total protein was harvested from cells transfected with p2A-wt at 6, 12, 18 and 24 h post-transfection. Protein lysates from cells transfected with the empty vector (Mock) or with the p2A-mut plasmid were harvested 24 h post-transfection. Western analysis was performed using a polyclonal antibody against eIF4GI. The positions of the molecular mass standards (in kDa) are shown. (**B**) COS-7 cells were transfected with plasmids p2A-wt or p2A-mut together with the dl HBZ 5′UTR plasmid and 24 h post-transfection. RLuc and FLuc activities were measured from cell lysates and expressed as RLA (%) to the activities obtained from the lysates expressing p2A-mut, which was set to 100% (+/– SEM). Values represent the mean (+/− SEM) for three independent experiments each conducted in duplicate. Statistical analysis was performed by an ANOVA test followed by a Dunnett’s multiple test comparison (*P < 0.05).

Next we evaluated the translational activity of the sHBZ 5′UTR in cells when eIF4G is cleaved by the HRV 2A protease, knowing that eIF4G cleavage by the HRV 2A protease hinders cap- but not IRES-dependent translation initiation (46,47). A plasmid expressing either a functional wild-type (p2A-wt) or an inactive mutant (p2A-mut) HRV protease (18,19) was transfected into COS-7 cells. Western analysis shows that eIF4G cleavage is observed starting at 12 h post-transfection (Figure 8A). However, no cleavage of eIF4G was observed in cells transfected with the p2A-mut at 24 h post-transfection (Figure 8A). Then either the p2A-mut or p2A-wt plasmids were co-transfected in cells with the dl sHBZ 5′UTR vector. The presence of HRV 2A protease (wt) reduced cap-dependent translation (RLuc) by 65%, while synthesis of the second cistron (FLuc) driven by the sHBZ 5′UTR was unaffected (Figure 8B). Thus, translation driven from the sHBZ 5′UTR was resistant to eIF4G cleavage by the HRV 2A protease in cells strongly suggesting that it promotes IRES-dependent initiation.

### Translation promoted by the sHBZ 5′UTR is dependent on ribosomal protein S25

**Figure 9.**
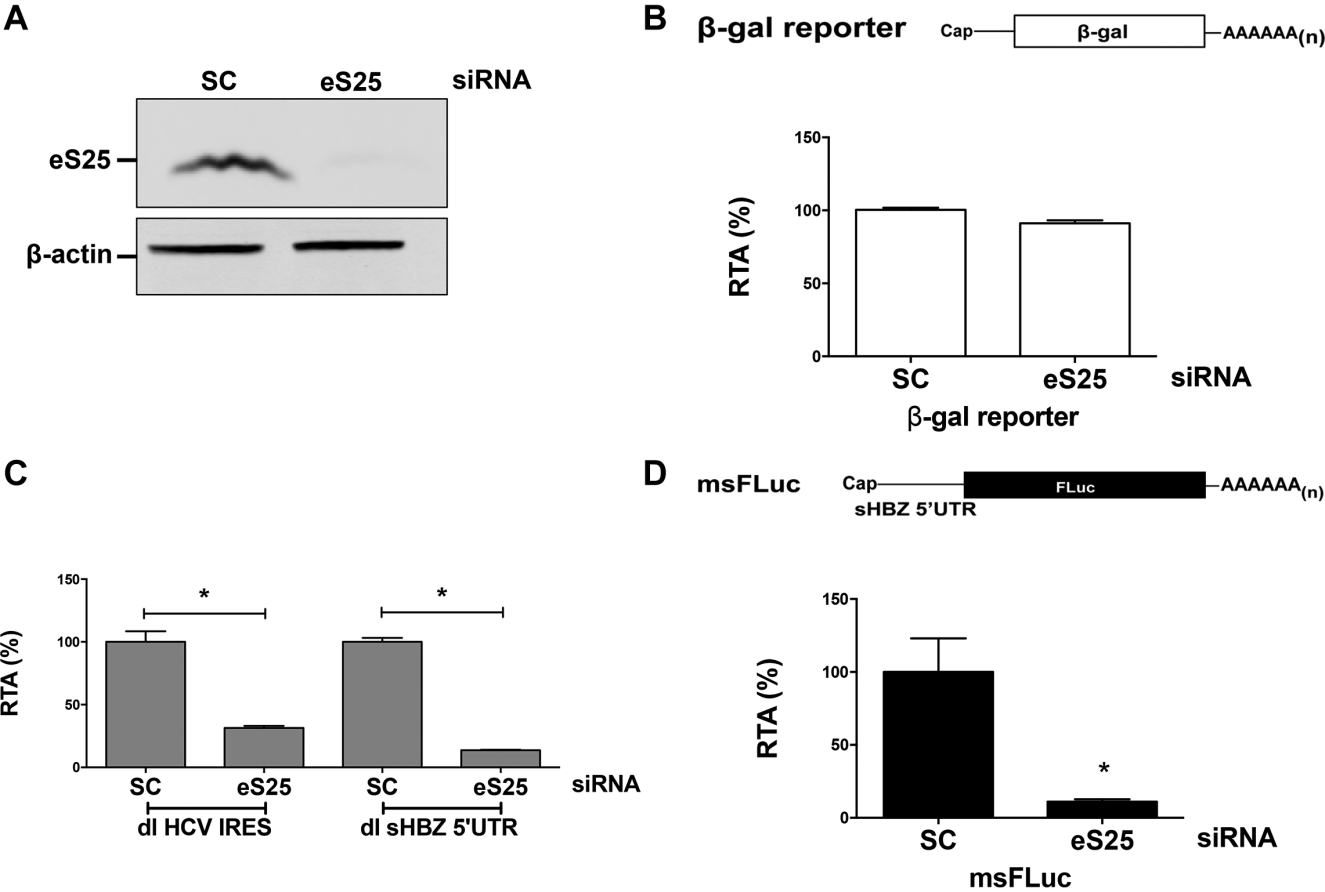
Translation mediated by the sHBZ 5′UTR requires eS25. COS-7 cells were transfected with a scrambled RNA or an siRNA that targeted eS25 mRNA together with either the msFLuc, the β- galactosidase, the dl sHBZ 5′UTR or the dl HCV IRES plasmids. Then 24 h post-transfection, protein lysates were harvested and subjected to western analysis (**A**), β-galactosidase assays (**B**) or luciferase assays (**C** and **D**) as indicated in ‘Materials and Methods’ section. (A) For western analysis, the membrane was probed with eS25 and β-actin antibodies and imaged using a LI-COR imager. (B) Effects on cap-dependent translation were measured by determining β-galactosidase activity expressed as RTA (%) relative to the scrambled control siRNA, which was set to 100%. Values are the mean (+/− SEM) for nine independent experiments, each performed in duplicate. (C) IRES activity from the dl sHBZ 5′UTR or the dl HCV IRES plasmids with or without eS25 was measured by determining the FLuc activity normalized to the transfection control β-galactosidase (FLuc/β-gal) and expressed as RTA (%) relative to the activity measured in cells transfected with the scrambled siRNA set to 100%. Values are the mean (+/− SEM) for three independent experiments, each performed in duplicate. Statistical analysis was performed by an ANOVA test followed by a Dunnett’s multiple test comparison (*P < 0.05). (D) The effect of eS25 knockdown on *in vitro* transcribed ^m7^G-capped msFLuc mRNA transfected into COS-7 cells, 48 h post siRNA knockdown was determined by measuring FLuc activity 4 h post-reporter transfection and expressed as RTA (%) relative to COS-7 cells transfected with the scrambled siRNA, which was set to 100%. Values are the mean (+/− SEM) for three independent experiments, each performed in duplicate. Statistical analysis was performed by a two-tailed *t*-test (*P < 0.05).

Viral IRESs, including the retroviral HIV-1 and HTLV-1 IRESs, rely on ribosomal protein S25 (eS25) for efficient translation (15,24,48,49). Importantly, cap-dependent translation initiation is not affected by the loss of eS25 (48,49). To determine if translation driven by the sHBZ 5′UTR required eS25 COS-7 cells were transiently transfected with an siRNA to knockdown eS25 or not (scrambled siRNA control; SC) (Figure 9A). Transfection of a plasmid that expressed a cap-dependent monocistronic mRNA (β-galactosidase; β-gal)) served as a control for transfection efficiency and cap-dependent translation (24,48), while the dl-HCV IRES plasmid served as a control of IRES-mediated translation initiation (48,49). As expected cap-dependent translation initiation was not impaired (Figure 9B), while the HCV IRES activity was reduced 69% (Figure 9C) when eS25 was knocked down. Translational activity driven by the sHBZ 5′UTR (dl sHBZ 5′UTR), decreased by 70% when eS25 was knocked down in COS-7 cells (Figure 9C), indicating that the activity of the sHBZ IRES is dependent on eS25.

Next, we sought to evaluate whether non-canonical translation mediated by the sHBZ 5′UTR contributes to the overall translational activity of the monocistronic capped mRNA, and if so, to what extent. For this, the msFLuc reporter plasmid was transfected into COS-7 cells that were knocked down for eS25 or not. As eS25 is required only for non-canonical initiation but not for cap-dependent translation initiation (Figure 9B and C and (48)), we reasoned that this strategy would uncouple cap-dependent from non-canonical translation initiation. FLuc expression from the msFLuc plasmid was reduced by 90% in cells that were knocked down for eS25 (Figure 9D). This finding suggests that translation initiation driven by the sHBZ 5′UTR, even when in the context of a monocistronic mRNA is mainly non-canonical (48), most probably IRES-dependent.

### Mapping the sHBZ IRES

**Figure 10.**
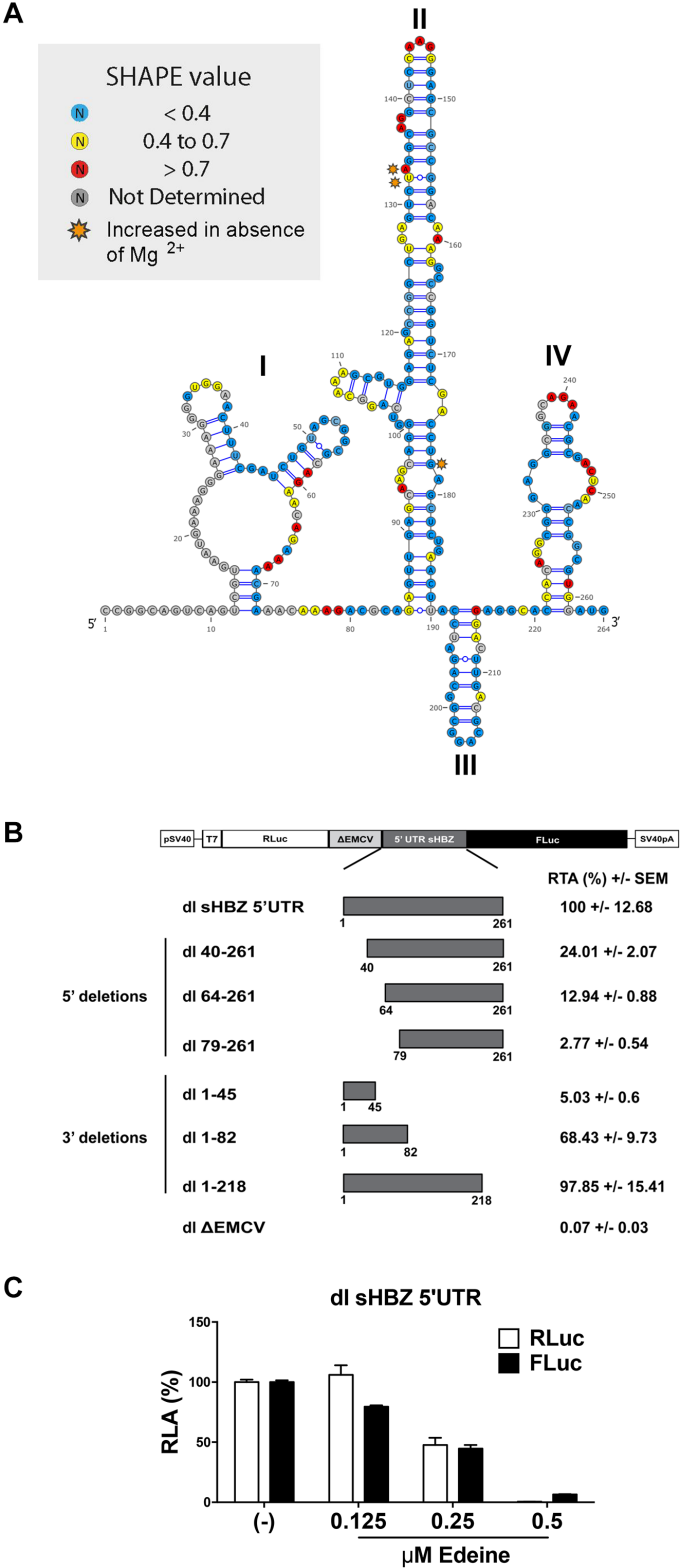
The sHBZ IRES maps to the 5′end of the 5′UTR. (**A**) Secondary structure model of the sHBZ 5′ UTR determined by RNA selective 2′ hydroxyl acylation analysis by primer extension (SHAPE) using 1-methyl-7-nitroisatoic anhydride (1M7) or N-methylisatoic anhydride (NMIA) as a modifying agents. The proposed structure is based on the mean SHAPE reactivity from three independent experiments conducted for each modifying reagent. The SHAPE reactivity values for each position using numbering according to the HTLV K30 infectious clone (genbank: L03561) on the sHBZ 5′UTR are indicated for each position on the HBZ 5′UTR according to the color code boxed, with decreased SHAPE reactivity <0.4 (blue), 0.4–0.7 (yellow), increased SHAPE reactivity >0.7 (red) and nucleotides not measured indicated in (gray). Nucleotides that exhibited an increased SHAPE reactivity to 1M7 or NMIA in the absence of Mg^2+^ are indicated (orange). (**B**) Dual luciferase plasmids containing the indicated 5′ and 3′ deletions were constructed and transfected into COS-7 cells and luciferase activity was measured 24 h later. The dl ΔEMCV vector was used as a negative control for IRES activity. The results are presented as RTA (%) relative to the dl sHBZ 5′UTR, set to 100%. Values shown are the mean (+/− SEM) for three independent experiments, each performed in duplicate. (**C**) The ^m7^G-capped *in vitro* transcribed dl sHBZ 5′UTR RNA reporters were translated in RRL in the absence (−) or the presence of increasing concentrations of edeine (0.125, 0.25 or 0.5 μM). RLuc and FLuc activities were measured and are shown as RLA (%) relative to the luciferase activity obtained in the absence (−) of edeine, set to 100%. Values shown are the mean (+/− SEM) for four independent experiments, each performed in duplicate.

To date, the structure of the sHBZ mRNA has not been investigated. We used SHAPE (selective 2′ hydroxyl acylation analysis by primer extension) (50) to map the structure of the sHBZ 5′UTR in solution using either 1-methyl-7-nitroisatoic anhydride (1M7) or N-methylisatoic anhydride (NMIA) as probes for ribose flexibility (Supplementary Figure S6). Structure probing was conducted in the presence or the absence of Mg^2+^ ions, as divalent cations favor tertiary structure motifs such as non-canonical base pairs or pseudoknots (Supplementary Figure S6). The sHBZ 5′UTR was mapped by comparing the primer extension profiles obtained from 1M7 or NMIA treated versus the untreated RNA. The two probes gave results that are consistent with a structured RNA (51,52), as reactivity profiles indicated that the vast majority of nucleotides were poorly reactive to either probing reagents (Supplementary Figure S6). Following an innovative workflow (37), we modeled sHBZ 5′UTR secondary structure taking into account both 1M7 and NMIA reactivity maps (Supplementary Figure S6) as well as phylogenetic data available for HTLV-1. Only a few isolated nucleotides appeared to be less reactive in presence of Mg^2+^ (orange stars in Figure 10A), this information did not allow us to detect any obvious pseudoknot interaction. However, differences observed between in the NMIA profiles in presence or absence of Mg^2+^ and with the 1M7 profile suggest the presence of non-canonical and tertiary interactions (52). The model for the secondary structure of the sHBZ 5′UTR composed of four (I–IV) stem loops domains (SLD) is shown in Figure 10A.

Based on the structural information, 5′ and 3′ deletions were generated to map the sHBZ IRES. Dual luciferase reporters containing 5′ and 3′ deletions (Figure 10B) were transfected into COS-7 cells. Removal of the first 39 nucleotides (nts) reduced IRES activity by 76%, while removal of the first 63 or 78 nts reduced the IRES activity 87 and 97%, respectively (Figure 10B). These observations indicate that SLD I (Figure 10A), plays an important role in sHBZ IRES activity. Deletions at the 3′ end of the 5′UTR affected IRES activity to a lesser extent (Figure 10B). The isolated region encompassing nts 1–45 had no IRES activity on its own. However, in agreement with the 5′ deletion mutants, 68% of the translational activity of the sHBZ IRES was harbored within SLD I, region comprising nts 1–82. Deletion of SLD IV (nts 219–261) had no significant effect on IRES activity (Figure 10B). Together, these results suggest that the main determinant of the sHBZ IRES is contained within the 5′end (nts 1–218) that is SLD I, SLD II and SLD III of the sHBZ 5′UTR.

Since SLD I contains 68% of the IRES activity, this suggests that the ribosome is recruited upstream of the initiation codon. This is consistent with our findings that the sHBZ IRES is sensitive to edeine in a monocistronic context (Figure 4). However, this has not been assessed in the context of a bicistronic mRNA. Therefore, the dl sHBZ 5′UTR RNA was *in vitro* translated in salt optimized RRL in the presence of increasing concentrations of edeine. As controls for this experiment the *in vitro* synthesized, capped dl PV IRES (harboring the poliovirus IRES), dl HCV IRES, dl HTLV-1 IRES and the dl HIV-1 IRES (harboring the HIV-1 IRES) bicistronic mRNAs were used. In agreement with previous reports (15,24,41), cap-dependent translation was reduced for all control bicistronic mRNAs in a drug concentration-dependent manner (Supplementary Figure S7). Translation initiation promoted by IRESs that are known to require 40S scanning (15), PV and HTLV-1 (Supplementary Figure S7A and B) was also sensitive to edeine. However, in conformity with previous reports the HCV and the HIV-1 IRESs (Supplementary Figure S7C and D) were not sensitive to edeine (24,41). Translation elongation is inhibited at 0.5 μM of edeine (Supplementary Figure S7) (41–43). *In vitro* translation of the dl sHBZ 5′UTR RNA was conducted in the presence of increasing concentrations of edeine. RLuc translation was inhibited at the lowest drug concentration (Figure 10C). IRES-mediated translation initiation from the dl sHBZ 5′UTR RNA was also inhibited by edeine (Figure 10C). This suggests that the sHBZ 5′UTR recruits a 40S subunit internally, which then scans down the mRNA in 5′to 3′ direction to reach the start codon.

### Activity of the sHBZ-IRES is dependent on eIF5A hypusination

**Figure 11.**
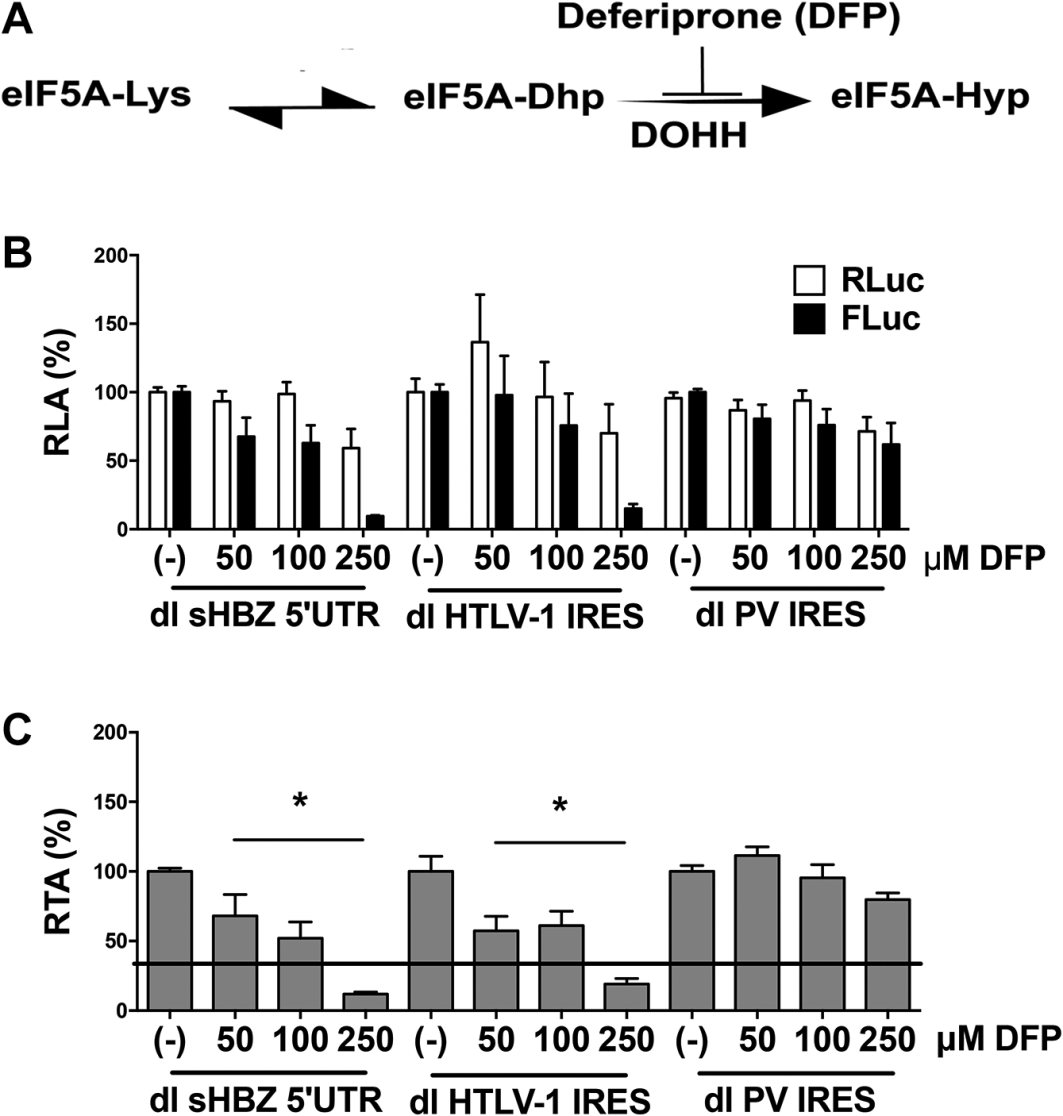
Translation initiation mediated by the sHBZ 5′UTR is reduced in the presence of deferiprone (DFP). (**A**) A diagram of the reaction for hypusine (Hyp) modification, which is added post-translationally to the eIF5A precursor by two consecutive enzymatic steps. First, the enzyme deoxyhypusine synthase catalyzes the transfer of a 4-aminobutyl moiety from the polyamine spermidine to a lysine residue in the eIF5A precursor to form the deoxyhypusine intermediate (eIF5A-Dhp). The enzyme DOHH then catalyzes the irreversible hydroxylation of Dhp to generate the hypusinated version of eIF5A (eIF5A-Hyp). As depicted, DOHH is inhibited by DFP. (**B** and **C**) COS-7 cells were co-transfected with either dl sHBZ 5′UTR, dl HTLV1 IRES or dl PV IRES and the transfection control pcDNA3.1-LacZ plasmid. Six hours post-transfection cells were treated with DFP (50, 100 or 250 μM). Total protein lysates were harvested 24 h post-transfection. (B) RLuc and FLuc activities were measured and normalized to β-galactosidase activity. Results are expressed as RLA (%), relative to the untreated control (−) set to 100%. Values shown are the mean (+/− SEM) of at least four independent experiments, each conducted in duplicate. (C) Data presented in (B) were used to determine the FLuc/RLuc ratio and data are presented as RTA (%), relative to the untreated control (−) set to 100%. Statistical analysis was performed by the ANOVA test followed by Dunnet multiple comparison (*P < 0.05).

Active eIF5A is hypusinated (Hyp) and acts as an IRES-transacting factor for several retroviral IRESs including the HTLV-1 IRES (23). To determine whether the sHBZ-IRES is dependent on active eIF5A, we evaluated the impact of deferiprone (DFP: 3-hydroxy-1,2-dimethylpyridin-4(1H)-one), a drug that blocks the activity of the enzyme deoxyhypusine hydroxylase (DOHH) (Figure 11A) (23) on sHBZ-IRES activity. The dl HTLV-1 IRES and the dl PV IRES plasmids were included as controls since the HTLV-1 IRES is dependent on active eIF5A, while the poliovirus IRES is not (15,23). As expected (23), the presence of DFP did not impact cap-dependent translation initiation, nor did it affect the activity of the PV IRES, while it inhibited the activity of the HTLV-1 and sHBZ IRESs (Figure 11B). This result indicates that the sHBZ-IRES relies on eIF5A-Hyp for its full activity. This conclusion is evident when data are presented as RTA where a significant decrease in the HBZ IRES activity is observed even in the presence of the lowest concentration (50 μM) of DFP (Figure 11C). Noteworthy, eIF5A also participated in the steps of translation elongation and termination (53), but since we have used reporters that encode for the exact same reporter proteins, we estimate that the main impact of DFP in our system is at the level of translation initiation.

## DISCUSSION

The HBZ proteins have multiple functions and highly relevant in HTLV-1 pathogenesis (2). Several reports suggest that the HTVL-1 antisense mRNAs that encode for the HBZ isoforms could be subject to translational control with a strong involvement of the 5′UTR in expressing the different isoforms of HBZ during HTLV-1 infection (4,5). Since translation is regulated at the initiation step and the 5′UTR plays a key role (9), we explored whether the 5′UTRs of the HBZ encoding mRNAs were responsible for the differential expression of the sHBZ and usHBZ isoforms. To study the function of the 5′UTRs without interference of any viral protein that could potentially modulate their function, the 5′UTRs of the *sHBZ* and *usHBZ* mRNAs were evaluated in the context of an exogenous mRNA in the absence of any virus or viral proteins both *in vitro* and in cells. Our results showed that differential expression of the sHBZ and usHBZ isoforms was regulated at the level of translation initiation (Figures 1 and 2). Translation from the 5′UTR of the *usHBZ* mRNA was inhibited by a yet undefined mechanism. Initially, we considered that the presence of multiple uAUGs present within the 5′UTR of the *usHBZ* mRNA could be responsible for inhibiting translation initiation (9); however, inhibition of protein synthesis was only marginally relieved by mutating the uAUGs present within the 5′UTR, never reaching the strength of the sHBZ 5′UTR (Figure 3). Further studies are required to fully understand the mechanisms regulating translation initiation mediated by the usHBZ 5′UTR. Nonetheless, from our data we can conclude that 40S ribosome recruitment is mediated by a cap-dependent mechanism and that recognition of the usHBZ initiation codon requires ribosome scanning (Figures 4, 5 and 6). In contrast, we showed that the sHBZ 5′UTR has an IRES, which recruits the 40S ribosomal subunit internally (Figures 5–10 and Supplementary Figures S2–S5). Translational activity of the sHBZ IRES was demonstrated in RRL (Figure 5 and Supplementary Figures S1–S4) and in mammalian cells (Figures 6–10 and Supplementary Figure S5). The sHBZ IRES resides within the 5′end (nts 1–218) of the sHBZ 5′UTR (Figure 10), which is consistent with the IRES using a scanning mechanism to recognize the initiation codon (Figure 10). Together, our results suggest that the sHBZ IRES employs a ‘land and scan’ mechanism of initiation, whereby the 40S subunit is recruited upstream of the start codon and scans in a 5′ to 3′ direction to the start codon (Figure 10). In addition, the activity of the sHBZ IRES is not hindered by eIF4G cleavage by the HRV 2A protease (Figure 8), but is dependent on ribosomal protein eS25 (Figure 9C). These latter findings are in full agreement with previous studies showing a role for eS25 in internal initiation (15,24,48,49). This observation alone indicates that translation initiation mediated by the 5′UTR of the *sHBZ* mRNA is mostly non-canonical (48), even in the context of a capped monocistronic mRNA (Figure 9D). In contrast to the *sHBZ* mRNA, translation initiation from the full-length mRNA of HTLV-1 and other retroviruses such as HIV-1 rely on both a canonical cap-dependent and a non-canonical IRES-dependent mechanisms of initiation (15,54).

The reason for why the capped-retroviral mRNAs, such as HIV-1 and HTLV-1 among others, harbor IRESs remains unknown (44). Furthermore, why a capped-mRNA such as that of sHBZ mostly relies on a cap-independent mechanism of translation initiation is unclear. However, the activity of most retroviral IRESs increases under conditions that are known to hinder cap-dependent translation initiation (44). In the case of the *sHBZ* mRNA, we can only speculate that its IRES assures protein expression during cellular stress induced by HTLV-1 replication. Upon infection, HTLV-1 enters into a latent state, rendering the infected individuals asymptomatic seropositive carriers, and only a small percentage of these carriers eventually develop ATL (55). Importantly, cellular stress conditions and cellular stress response mechanisms activate latent HTLV-1 by stimulating transcription of viral mRNA (56,57). Upon HTLV-1 activation viral accessory proteins directly participate in the maintenance and further stimulation of a cellular stress, an environment required for HTLV-1 replication (58). HTLV-1 replication itself induces additional conditions known to negatively impact canonical translation initiation, for example, cleavage of eIF4G and inhibition of canonical cap-dependent translation by the HTLV-1 protease (59). Herein, we show that sHBZ IRES activity is resistant to eIF4G cleavage by a viral protease (Figure 8). These data suggest that the expression of the HBZ protein would be maintained during HTLV-1-induced cleavage of eIF4G. HTLV-1 replication also induces the generation of reactive oxygen species (58), a condition that is required for enhanced expression of viral proteins (60,61). Studies suggest that increase in viral protein expression under cellular stress conditions is regulated mainly at the level of mRNA translation (60,61). Notably, in contrast to cap-dependent translation that is hindered under stress conditions, IRES-mediated translation initiation is in general promoted (11,12).

ATL is a fatal disease resistant to most chemotherapeutic agents (1,62) and HBZ is important in maintaining the leukemic state in ATL patients (63,64). Notably, proliferation of ATL cells is stimulated when HBZ is overexpressed and hindered when the expression of HBZ is suppressed (6). Furthermore, silencing of HBZ decreases the ability of HTLV-1 infected T cells to form solid tumors in mice (65). Due to this and other roles played by HBZ during HTLV-1 replication and pathogenesis (2), the protein and its mRNAs have been envisioned as a potential target for the development of novel therapeutic approaches to treat HTLV-1-related illnesses (66). An important finding of this study is that IRES-mediated translation initiation is the main mechanism used by the sHBZ 5′UTR to drive protein synthesis. Importantly, IRES-mediated translation initiation has been recognized, as a potential target for the development of novel therapeutics designed to specifically inhibit non-canonical mechanisms of translation initiation (67). In support of this possibility herein, we show that by knocking down ribosomal protein eS25, sHBZ IRES activity can be reduced, without impacting cap-dependent translation initiation (Figure 9). This finding suggests that by targeting a subset of cellular proteins required for IRES activity the expression from the s*HBZ* mRNA can be hindered. Furthermore, it confirms that sHBZ IRES activity can be targeted in order to inhibit viral gene expression. In addition, we show that when used in clinical doses DFP, an oral iron chelator used in the clinic for the treatment of iron overload (68) inhibits HBZ IRES-mediated translation initiation in cells (Figure 11). Therefore, our results provide strong evidence favoring the notion that cap-independent translation initiation of *sHBZ* mRNA can be envisioned as a specific target for the development of novel antiviral therapies. As shown herein, in-depth knowledge of the molecular mechanisms driving HBZ expression and sHBZ IRES activity in particular might prove paramount not only for the understanding HTLV-1 pathogenesis, but also for the identification of novel strategies to exploit in order to treat and prevent viral induced diseases by targeting HBZ protein synthesis.

## Supplementary Material

Supplementary Data
