# Supplementary material for: Non-canonical translation initiation of the spliced mRNA encoding the human T-cell leukemia virus type 1 basic leucine zipper protein
Source: Nucleic Acids Res. 2018 Sep 12;46(20):11030–47. doi: 10.1093/nar/gky802 (PMC6237760; doi:10.1093/nar/gky802)
Supplement: Supplementary Data [file gky802_supplemental_files.pdf]

SUPPLEMENTAL FIGURE and FIGURE LEGENDS.

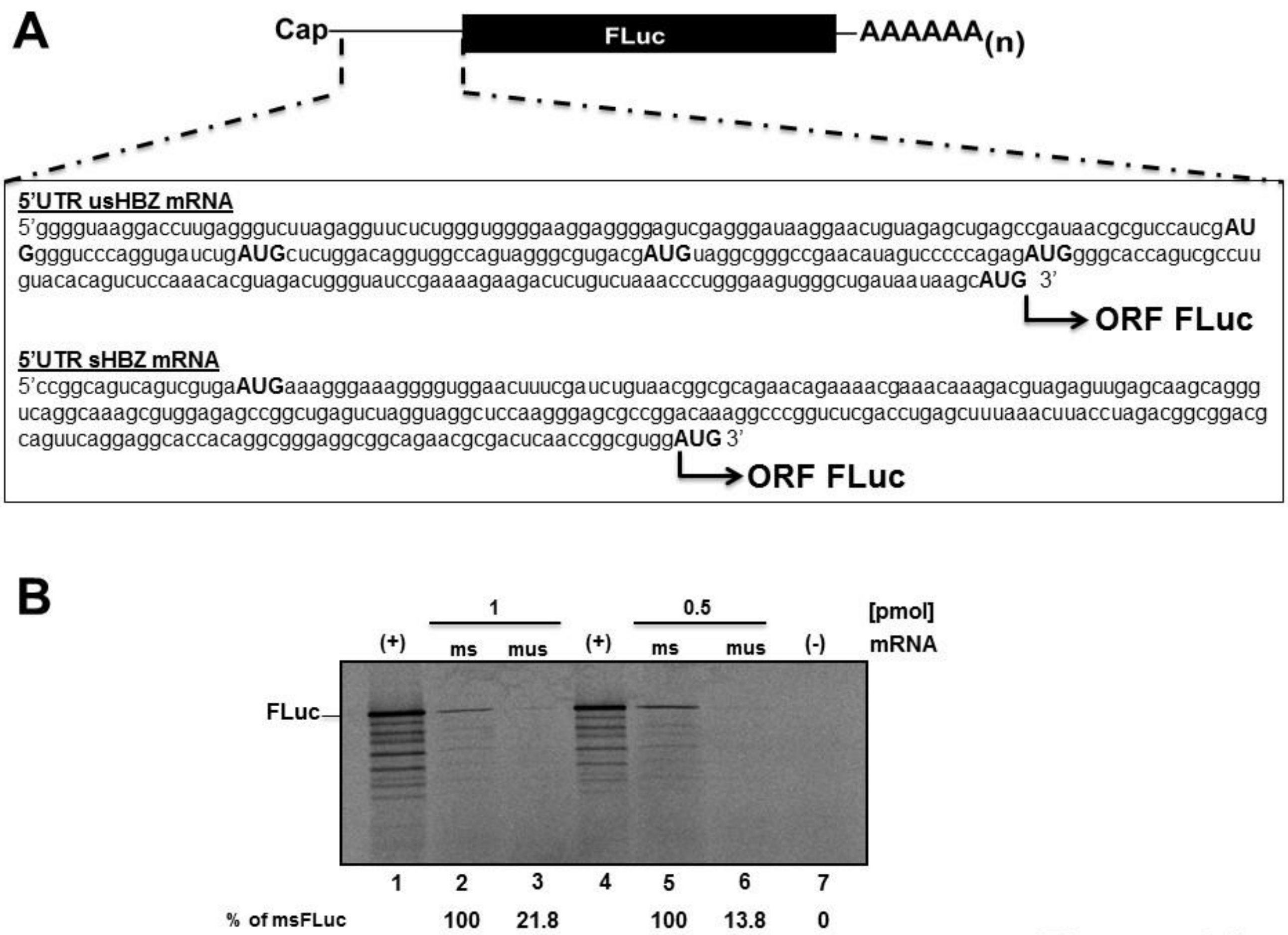

Figure S1

Supplemental Figure S1. The HBZ 5'UTR. (A) Nucleotide sequence of the 5'UTR of the *usHBZ* and *sHBZ* mRNAs in the context of the capped and polyadenylated monocistroinic mRNA encoding firefly luciferase (FLuc) used in the assays. The musFLuc mRNA harbors the 5'UTR of the *usHBZ* mRNA upstream of the FLuc open reading frame (ORF). The msFLuc mRNA harbors the 5'UTR of the *sHBZ* mRNA upstream of FLuc. RNAs differ exclusively in their 5'UTR. The upstream AUGs (uAUGs) as well as the FLuc initiation codon are indicated in bold capital letters. **(B)** The *in vitro* transcribed capped and polyadenylated musFLuc or msFLuc mRNAs were *in vitro* translated in RRL in the presence of ^{35}S-methionine (^{35}S-met) and proteins were resolved by SDS–PAGE (12%) (1). As a positive control for the expression of the FLuc protein, an FLuc control mRNA supplied with the RRL kit was used (lanes 1 and 4). The labeled products were visualized and quantified using a Storm PhosphorImager (GE Healthcare). The quantified labeled FLuc product is expressed as percentage (%) relative to FLuc generated by of msFLuc that was set to 100 %.

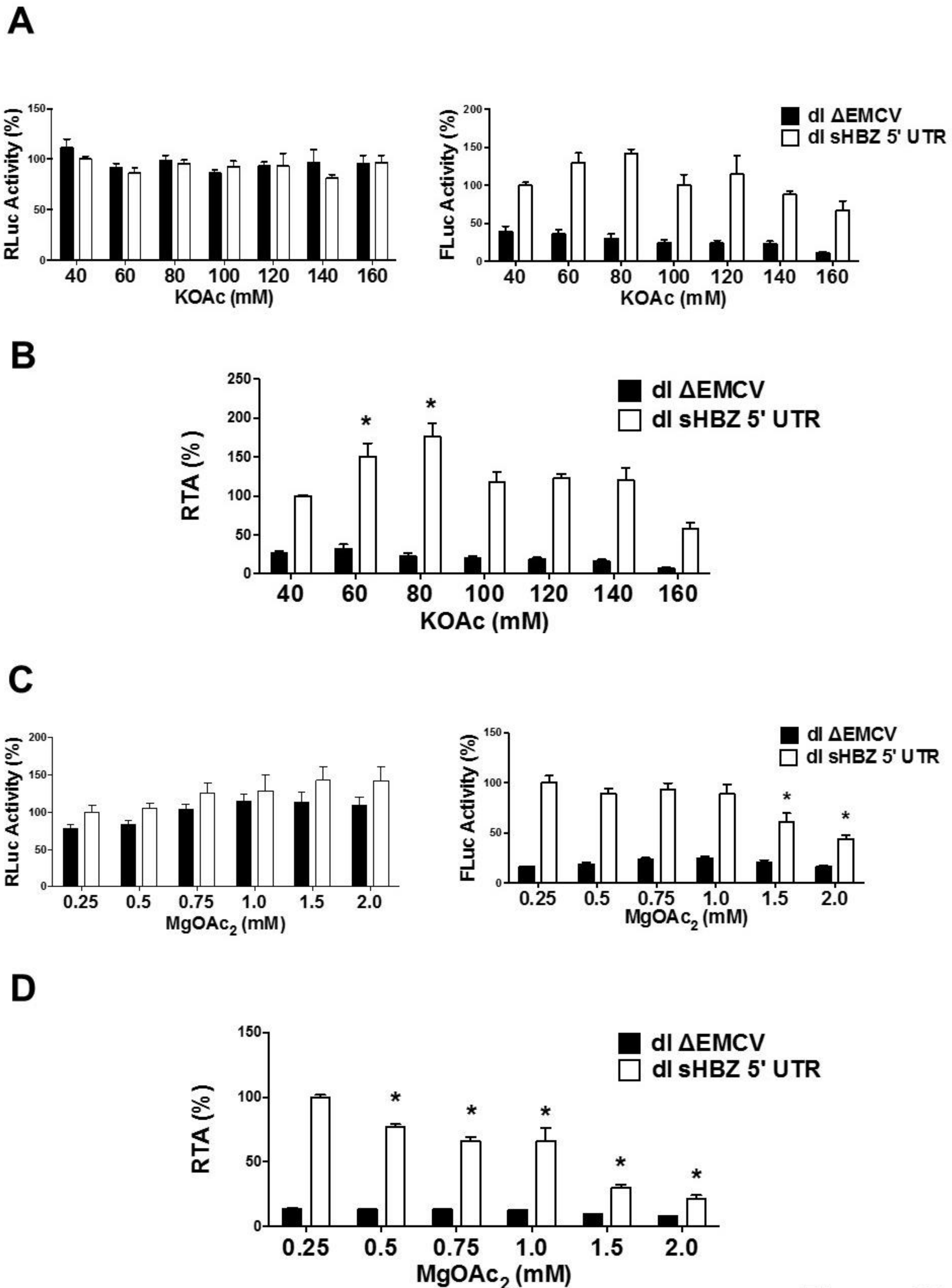
A
RLuc Activity (%)
FLuc Activity (%)
KOAc (mM)
dl ΔEMCV
dl sHBZ 5' UTR
B
RTA (%)
KOAc (mM)
dl ΔEMCV
dl sHBZ 5' UTR
C
RLuc Activity (%)
FLuc Activity (%)
MgOAc2 (mM)
dl ΔEMCV
dl sHBZ 5' UTR
D
RTA (%)
MgOAc2 (mM)
dl ΔEMCV
dl sHBZ 5' UTR

Figure S2

Supplemental Figure S2. Optimization of potassium and magnesium acetate concentration in RRL for translation driven by the sHBZ 5'UTR. The dl sHBZ 5'UTR (white bars) or dl ΔEMCV (black bars) capped RNAs were translated in RRL in the presence of varying concentrations of KOAc **(A and B)** or $MgOAc_2$ **(C and D)**. Values are shown as RLA (%) (A and C) or as RTA (%) (B and D) with the RLuc and FLuc activities shown relative to RRL without additional salt supplementation (0.25 mM $MgOAc_2$ and 40 mM KOAc), which was set to 100 %. Values shown are the mean (+/- SEM) for three independent experiments, each performed in duplicate. Statistical analysis was performed by an ANOVA test followed by a Dunnett's multiple test comparison (*P < 0.05).

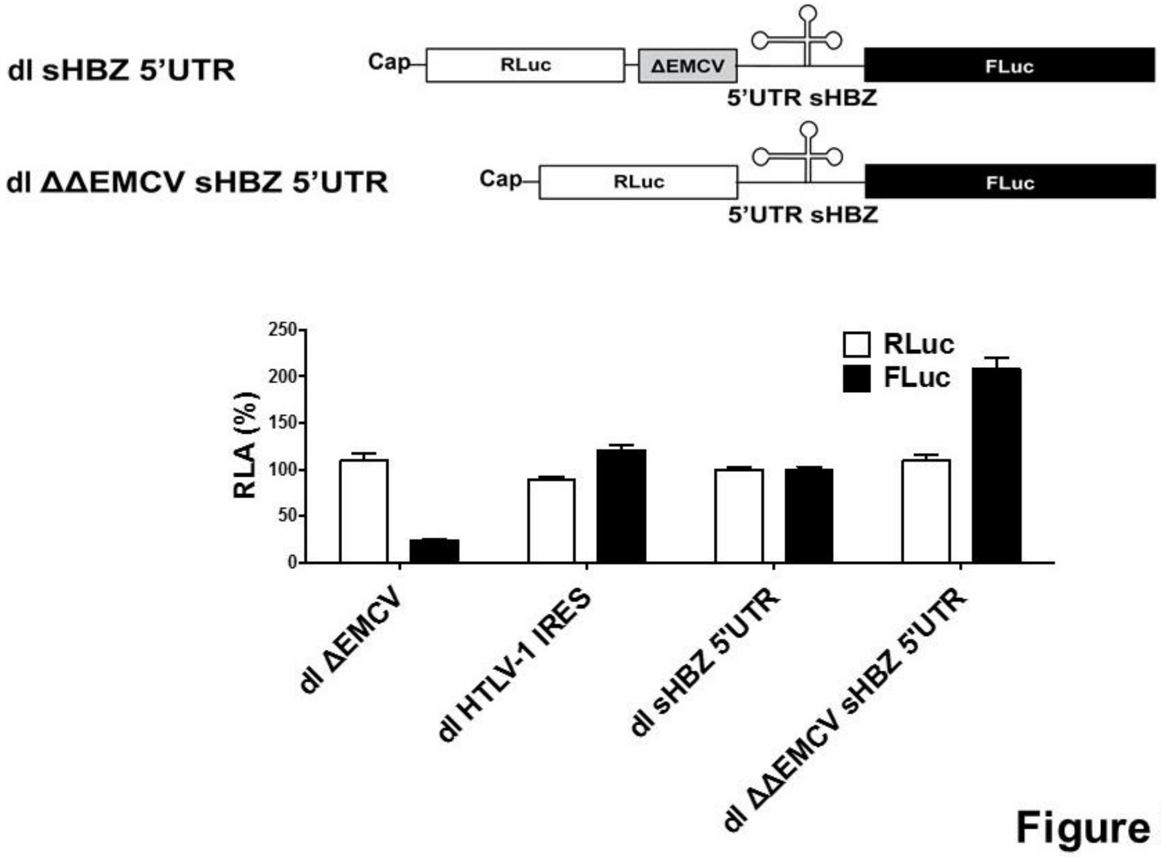

Figure S3

Supplemental Figure S3. Translation promoted by the sHBZ 5'UTR in RRL is independent of the ΔEMCV sequences. The upper panel depicts a schematic representation of the in vitro transcribed dl RNAs used in the *in vitro* translation studies. The ^{m7}G-capped dl sHBZ 5'UTR RNA, containing ΔEMCV element, or the dl ΔΔEMCV sHBZ 5'UTR RNA were translated in salt optimized RRL and luciferase activity was measured. The RLuc (white bars, lower panel) and FLuc (black bars, lower panel) activities are shown relative to the dl sHBZ 5'UTR RNA, which was set to 100%. Values shown are the mean (+/- SEM) for three independent experiments, each performed in duplicate.

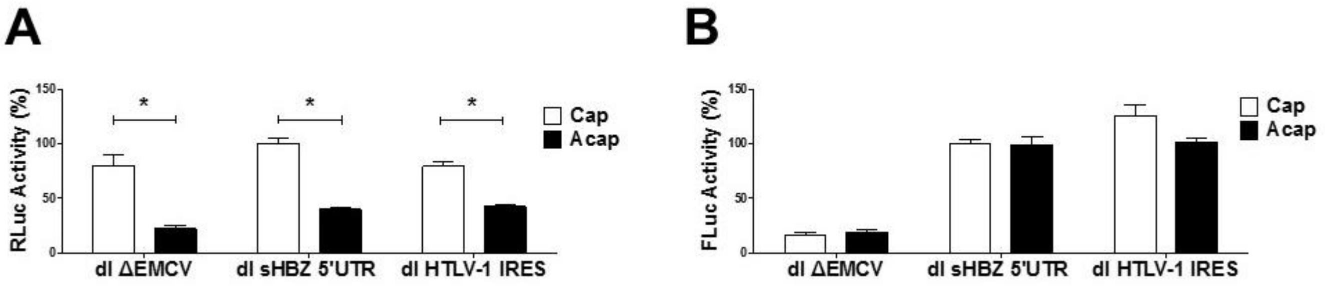

Figure S4

Supplemental Figure S4. Translation promoted by the sHBZ 5'UTR in RRL is independent of the ^{m7}G-cap structure. Salt optimized RRL was programmed with *in vitro* transcribed RNA harboring either a functional 5'm7GpppG (cap) or an ApppG cap-analog (Acap). Results are presented as RLA with the RLuc **(A)** and FLuc **(B)** activities relative to RRL programmed with the capped dl sHBZ 5'UTR mRNA, which was set to 100 %. Values are the mean (+/- SEM) for three independent experiments, each performed in duplicate. Statistical analysis was performed by a two-tailed t-test (*P < 0.05) between each capped and Acapped RNA with the same intercistronic sequence.

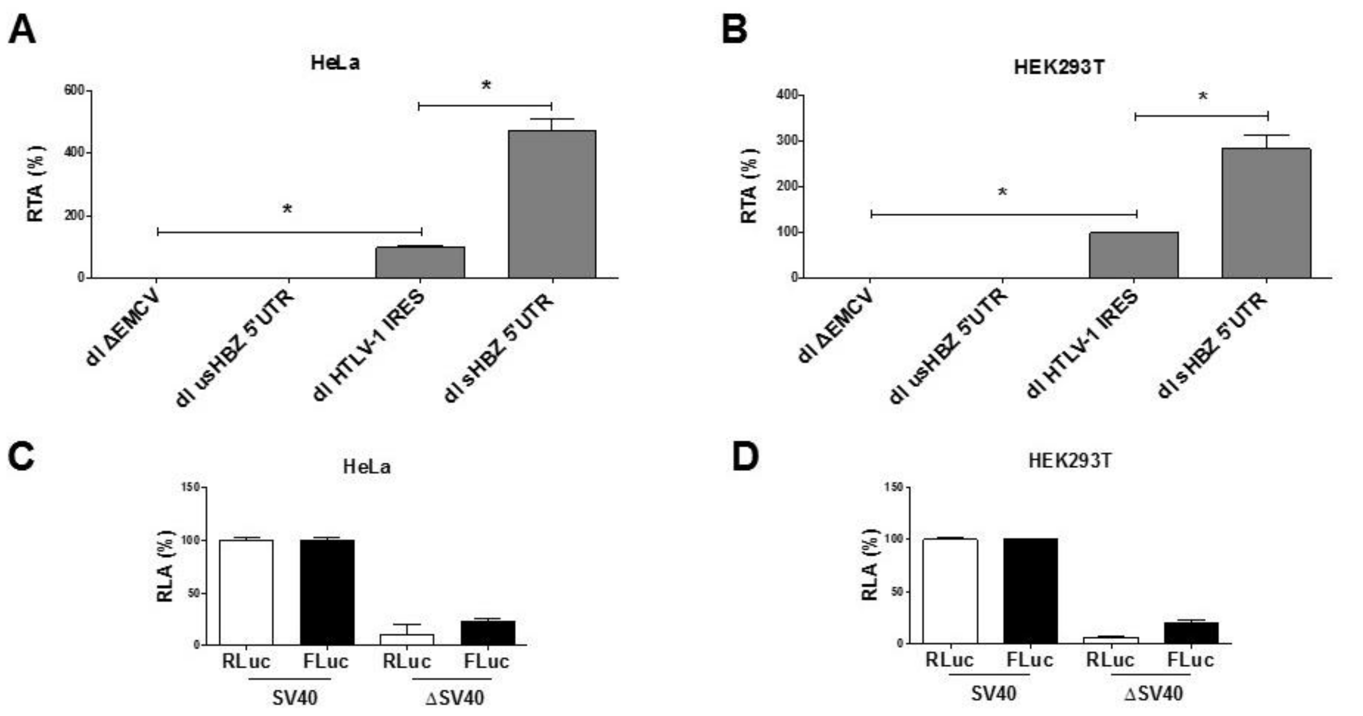

Figure S5

Supplemental Figure S5. The sHBZ 5'UTR has IRES activity in HeLa and HEK 293T cells. The dl ΔEMCV, dl HTLV-1 IRES, dl usHBZ 5'UTR or dl sHBZ 5'UTR plasmids were co-transfected into HeLa **(A)** or HEK 293T **(B)** cells with the pcDNA3.1 *lac*Z transfection control plasmid (2,3). RLuc and FLuc activities were measured 24 h after transfection and normalized to the β-galactosidase activity and expressed as RTA (%) relative to the dl HTLV-1 IRES, which was set to 100%. Values are the mean (+/- SEM) for three independent experiments, each performed in duplicate. Statistical analysis was performed by an ANOVA test followed by a Dunnett's multiple test comparison (*P < 0.05). HeLa **(C)** or HEK 293T **(D)** cells were co-transfected with the dl sHBZ 5'UTR or the ΔSV40 dl sHBZ 5'UTR plasmids and the pcDNA3.1 *lac*Z transfection control plasmid. RLuc and FLuc activities were measured 24 h after transfection and normalized to the β-galactosidase activity. Values are expressed as RLA (%) relative to the dl sHBZ 5'UTR, which was set to 100%. Values are the mean (+/- SEM) for three independent experiments, each performed in duplicate.

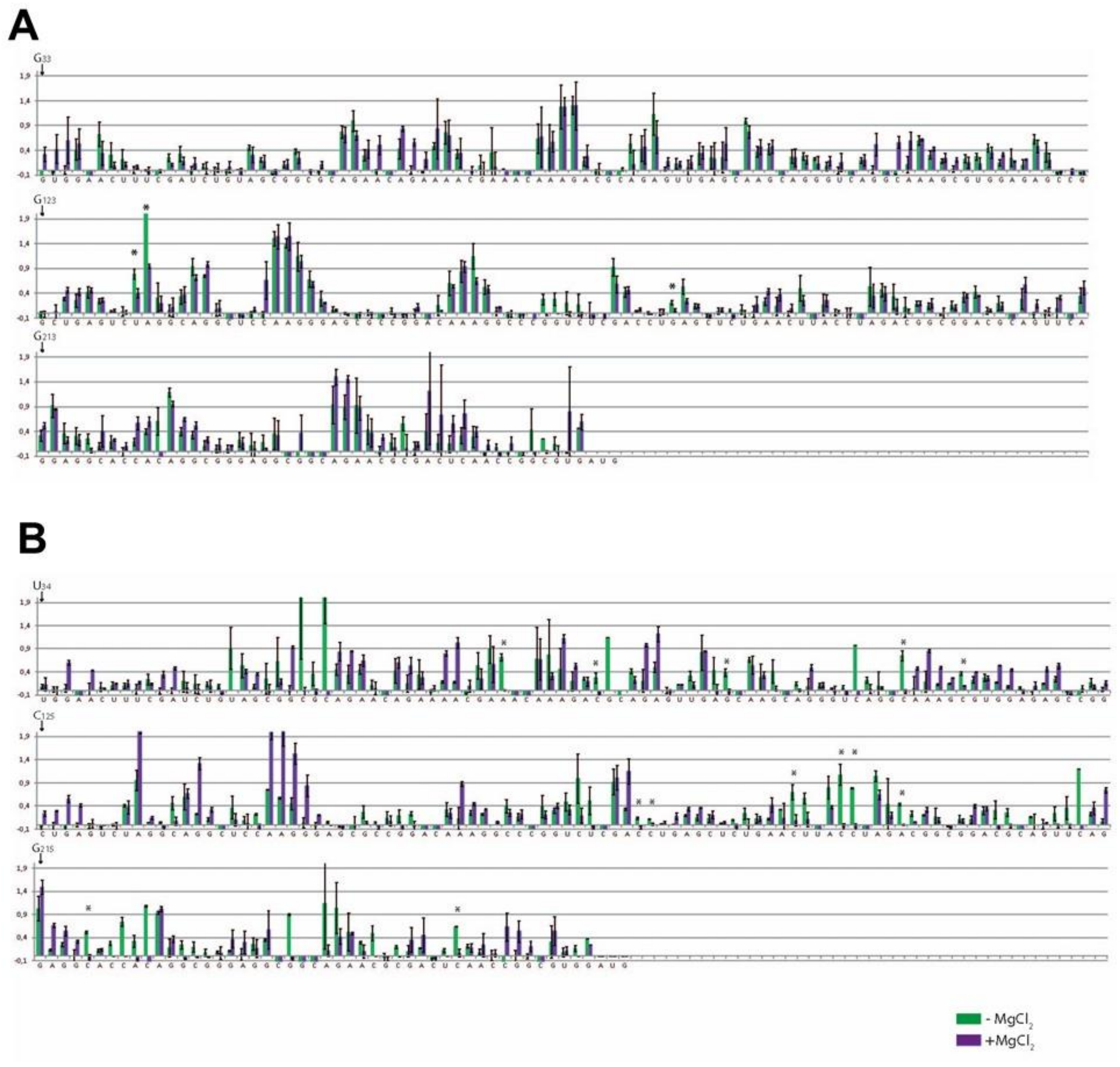

Figure S6

Supplemental Figure S6. SHAPE reactivity profile. RNA selective 2' hydroxyl acylation analysis by primer extension (SHAPE) was conducted using 1-methyl-7-nitroisatoic anhydride (1M7) (A) or N-methylisatoic anhydride (NMIA) (B) as a modifying agents as previously described (4-7). The 1M7 and NMIA reactivity profiles were obtained in the absence (green, solid) or presence (purple, solid) of $MgCl_2$. Relevant nucleotide positions are indicated (arrows). Error bars are the standard error for at least three independent experiments.

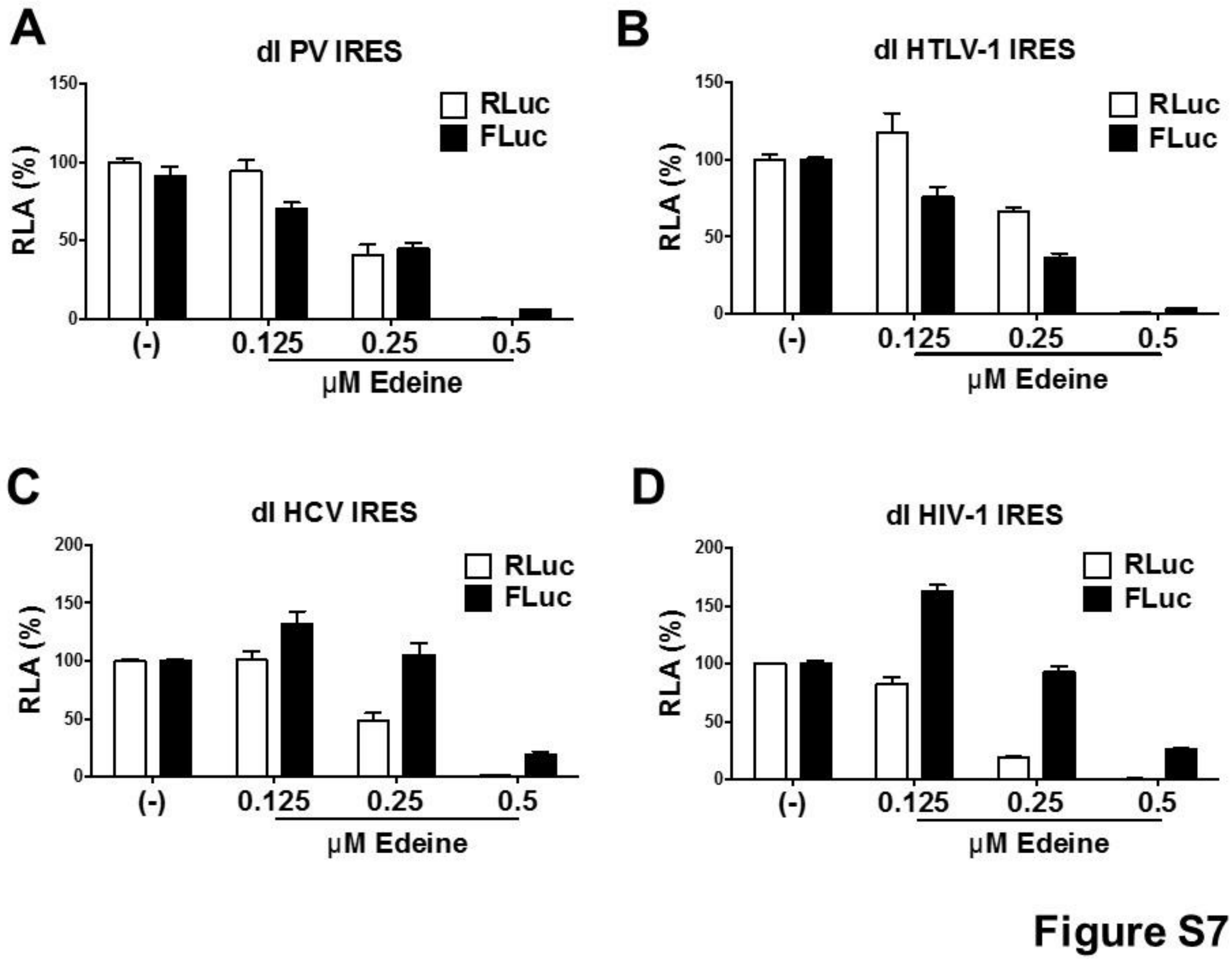

Supplemental Figure S7. Translation initiation of bicistronic mRNAs in the presence of edeine. The ^{m7}G-capped dl PV IRES **(A)**, dl HTLV-1 IRES **(B),** dl HCV IRES **(C),** or dl HIV-1 IRES **(D)** *in vitro* transcribed RNAs were translated in RRL in the absence (-) or presence of increasing concentrations of edeine (0.125, 0.25, or 0.5 µM). RLuc (white bars) and FLuc (black bars) activities were measured and are shown relative to the luciferase activity obtained in the absence (-) of edeine, set to 100%. Values shown are the mean (+/- SEM) for four independent experiments, each performed in duplicate.

REFERENCES SUPPLEMENTAL FIGURES.

1. Angulo, J., Ulryck, N., Deforges, J., Chamond, N., Lopez-Lastra, M., Masquida, B. and Sargueil, B. (2016) LOOP IIId of the HCV IRES is essential for the structural rearrangement of the 40S-HCV IRES complex. *Nucleic acids research*, **44**, 1309-1325.
2. Carvajal, F., Vallejos, M., Walters, B., Contreras, N., Hertz, M.I., Olivares, E., Caceres, C.J., Pino, K., Letelier, A., Thompson, S.R. *et al.* (2016) Structural domains within the HIV-1 mRNA and the ribosomal protein S25 influence cap-independent translation initiation. *The FEBS journal*, **283**, 2508-2527.
3. Olivares, E., Landry, D.M., Caceres, C.J., Pino, K., Rossi, F., Navarrete, C., Huidobro-Toro, J.P., Thompson, S.R. and Lopez-Lastra, M. (2014) The 5' untranslated region of the human T-cell

lymphotropic virus type 1 mRNA enables cap-independent translation initiation. *Journal of virology*, **88**, 5936-5955.
4. Chamond, N., Deforges, J., Ulryck, N. and Sargueil, B. (2014) 40S recruitment in the absence of eIF4G/4A by EMCV IRES refines the model for translation initiation on the archetype of Type II IRESs. *Nucleic Acids Res*, **42**, 10373-10384.
5. Deforges, J., Chamond, N. and Sargueil, B. (2012) Structural investigation of HIV-1 genomic RNA dimerization process reveals a role for the Major Splice-site stem loop. *Biochimie*, ***94***, 1481-1489.
6. Mortimer, S.A. and Weeks, K.M. (2007) A fast-acting reagent for accurate analysis of RNA secondary and tertiary structure by SHAPE chemistry. *J Am Chem Soc*, **129**, 4144-4145.
7. Wilkinson, K.A., Gorelick, R.J., Vasa, S.M., Guex, N., Rein, A., Mathews, D.H., Giddings, M.C. and Weeks, K.M. (2008) High-throughput SHAPE analysis reveals structures in HIV-1 genomic RNA strongly conserved across distinct biological states. *PLoS Biol*, **6**, e96.
